# Exploring human mixing patterns based on time use and social contact data and their implications for infectious disease transmission models

**DOI:** 10.1186/s12879-022-07917-y

**Published:** 2022-12-19

**Authors:** Thang Van Hoang, Lander Willem, Pietro Coletti, Kim Van Kerckhove, Joeri Minnen, Philippe Beutels, Niel Hens

**Affiliations:** 1 I-Biostat, Data Science Institute, Hasselt University, Martelarenlaan 42, 3500 Hasselt, Belgium; 2 Centre for Health Economic Research and Modelling Infectious Diseases, Vaccine & Infectious Diseases Institute, University of Antwerp, Universiteitsplein 1, 2610 Antwerp, Belgium; 3 Vrije Universiteit Brussel, Brussel, Belgium; 4 School of Public health and Community Medicine, University of New South Wales, 2052 Sydney, Australia

**Keywords:** Infectious disease dynamics, Mixing patterns, Exposure matrices, Spatial dynamics, Time use

## Abstract

**Background:**

The increasing availability of data on social contact patterns and time use provides invaluable information for studying transmission dynamics of infectious diseases. Social contact data provide information on the interaction of people in a population whereas the value of time use data lies in the quantification of exposure patterns. Both have been used as proxies for transmission risks within in a population and the combination of both sources has led to investigate which contacts are more suitable to describe these transmission risks.

**Methods:**

We used social contact and time use data from 1707 participants from a survey conducted in Flanders, Belgium in 2010–2011. We calculated weighted exposure time and social contact matrices to analyze age- and gender-specific mixing patterns and to quantify behavioral changes by distance from home. We compared the value of both separate and combined data sources for explaining seroprevalence and incidence data on parvovirus-B19, Varicella-Zoster virus (VZV) and influenza like illnesses (ILI), respectively.

**Results:**

Assortative mixing and inter-generational interaction is more pronounced in the exposure matrix due to the high proportion of time spent at home. This pattern is less pronounced in the social contact matrix, which is more impacted by the reported contacts at school and work. The average number of contacts declined with distance. On the individual-level, we observed an increase in the number of contacts and the transmission potential by distance when travelling. We found that both social contact data and time use data provide a good match with the seroprevalence and incidence data at hand. When comparing the use of different combinations of both data sources, we found that the social contact matrix based on close contacts of at least 4 h appeared to be the best proxy for parvovirus-B19 transmission. Social contacts and exposure time were both on their own able to explain VZV seroprevalence data though combining both scored best. Compared with the contact approach, the time use approach provided the better fit to the ILI incidence data.

**Conclusions:**

Our work emphasises the common and complementary value of time use and social contact data for analysing mixing behavior and analysing infectious disease transmission. We derived spatial, temporal, age-, gender- and distance-specific mixing patterns, which are informative for future modelling studies.

**Supplementary Information:**

The online version contains supplementary material available at 10.1186/s12879-022-07917-y.

## Background

Infectious diseases have substantial impact on public health and economy and warrant constant monitoring and follow-up. Initially, transmission models relied on untested assumptions about “at risk events”. In recent years, disease transmission models have been informed by social contact surveys as, e.g., those obtained from the large-scale European POLYMOD project [1], in which participants had to report about their contact behavior by age, gender, frequency, etc. Social contact patterns have been successfully used as proxies for transmission of close-contact diseases, such as influenza and mumps, under the social contact hypothesis [2]. The use of social contact data helps estimating key epidemiological parameters  [3, 4], behavioral changes [5–7] and demographic change [8] in the context of disease transmission. The number of social contact surveys to collect empirical data on human contact behavior has increased substantially over recent years [9].

Next to social contact data, also time use data have proven their value for explaining infectious disease data using the *time use approach* in which mixing patterns can be estimated from the time spent at a given location [10–12]. The reported presence over time at different locations during a day enables the estimation of the exposure time among age groups  [12]. The notion of co-presence is complementary to reported social contacts, hence time use data are useful to capture “at risk events” that fall outside the definition of a social contact.

The integration of both the “time use approach” and the “social contact approach” has lead to estimation of “suitable contacts”  [11]. De Cao et al. [11] showed that the interplay between exposure time and social contacts appeared to be important to study the transmission of Varicella-Zoster virus (VZV) whereas the transmission dynamics of parvovirus-B19 was best captured using only exposure time. This study was based on two independently collected surveys (social contacts and time use).

A systematic review of social contacts surveys [9] revealed that only a limited number of contact studies investigated the relationship between contacts and distance whereas next to the number of contacts, contact dispersal is of essence to capture disease transmission dynamics. Disease counts have been modeled using power law dispersal kernels [13–15], though questions remain whether this also holds for social mixing behavior. A study in Great Britain [16] collected information on the distance from home for each contact and observed a decrease in contact duration with increasing distance from home. In addition, age-specific contact patterns and temporal differences with respect to weekdays and weekends have been described [9], but gender-specific behavior in social mixing is less reported, though could contribute to the parameterisation of mathematical transmission models [17, 18].

In this work, we use data from one single survey to compare the social contact, time use and suitable contact approach. An important element in this survey [18] is the recording of the distance from home for every reported location in the time use survey. In particular, we (1) compared mixing patterns based on time use and social contact data, and explored covariates such as age, gender, location, etc.; (2) analysed social mixing patterns and estimated basic reproduction numbers by distance from home; (3) evaluated the value of time use and social contact data sources to explain seroprevalence data for VZV and parvovirus-B19, and influenza-like illness (ILI) incidence data in Belgium.

## Methodology

**Fig. 1:**
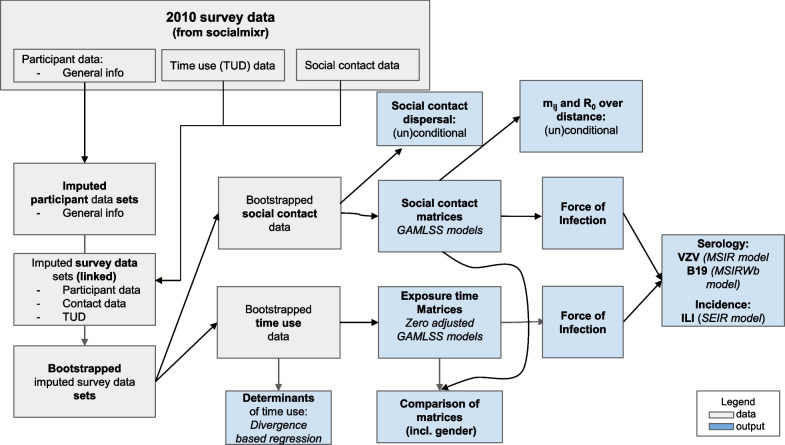
Flowchart of the methodology

This analysis is based on three types of data: social contacts, time use, and clinical information w.r.t. respiratory diseases. We first introduce each dataset and continue with the description of our analysis starting from our methods to handle missing data and uncertainty. Next, we describe how we analysed exposure time and social contact data and calculated mixing matrices. We end with the integration and evaluation of mixing patterns in models for VZV, parvovirus-B19 and ILI incidence data from Belgium. We present the overview of the methodology in Fig. 1 followed by the detailed description of the methods used in the sub-sections below.

### Data

This study is based on a diary-based survey on social contacts and time use, which was conducted between September 2010 and February 2011 in Flanders, Belgium. Two types of contacts were defined: (1) two-way conversations during which at least three words were spoken and (2) contacts that involved skin-to-skin touching. Information recorded in the diary included sex and the exact age or presumed age interval of each contacted person over the entire day. More information on this data set can be found in [18] and the dataset is available online within the social contact data sharing initiative [19] and the SOCRATES platform [20].

To record time use, participants were asked to indicate for predefined time slots the location at which they spent most of their time. Location types were pre-defined (home, kindergarten, school, workplace, transport, family, leisure and other) and the options were adapted to the age of the participant. For example “kindergarden” was only used for surveying children and “workplace” only for adults. We also questioned the distance from home for each location in four categories (0–1 km, 2–9 km, 10–74 km or 75 km or more). Time slots were mostly of 1-h length, except in the morning (2–5 h, 5–8 h) and in the evening (20–22 h, 22–24 h, 24 h–2 h). With respect to missing data, 1486 participants (87%) provided full information on their location and distance from home for each time-slot, and 49 participants provided no time use data at all. The final sample size used for the analysis in this work is 1707 participants, as in [18].

Seroprevalence data was used from the Belgian sero-survey in 2001–2003 on parvovirus-B19 and VZV. In total, 3080 sera were tested for parvovirus-B19 and 3256 sera were tested for VZV, from which 2975 sera were tested for both VZV and parvovirus-B19. The sero-survey involved participants ranging from 0 to 71.5 years of age. More information is provided in Hens et al [21].

The ILI incidence data were collected from a network of general practitioners (GPs) in Belgium. The GPs in the network were asked to report the weekly number of ILI by 7 age groups: <1, 1–4, 5–14, 15–19, 20–64, 65–84, $\ge$ 85 years. The data was provided by the Scientific Institute of Public health. More details of the data are described in [22, 23].

### Analysis

#### Missing data

Missing values in the time use data were treated as “Missing at Random” [24]. As such, we assumed a systematic relationship between the propensity of missing values and the observed data, but not with the missing data. Missing data were imputed by using Multivariate Imputation via Chained Equations (MICE) [25]. The list of variables included in the imputation model can be found in Additional file 1: Table S3. By applying MICE with different random number generator seeds, we obtained 10 imputed datasets. Please note that for the initial data exploration (Table 1), as described in the first part of the Results section, we used the original data with location type set to “Missing”.

**Table 1:** Reported daily time use by location with respect to age, gender, type of day, period, health state, household type

Covariate	N (%)	Home (%)	School (%)	Work (%)	Transport (%)	Other (%)	Missing (%)
Age	**1707**						
[0–3)	87 (5.10)	77.82	8.53	0	2.06	9.44	2.15
[3–6)	84 (4.92)	74.51	11.96	0	2.88	9.33	1.32
[6–12)	125 (7.32)	64.50	15.30	0	2.60	13.24	4.36
[12–18)	79 (4.63)	64.72	11.66	1.11	4.43	15.88	2.20
[18–25)	99 (5.80)	58.67	7.45	9.47	4.71	16.46	3.24
[25–45)	524 (30.70)	61.86	0.29	16.9	4.18	13.19	3.58
[45–65)	468 (27.42)	62.36	0.20	12.77	4.18	13.79	6.70
65+	241 (14.11)	76.31	0	0.48	4.26	11.50	7.45
Gender	**1707**						
Female	917 (53.72)	66.34	2.82	8.33	3.72	13.72	5.07
Male	790 (46.28)	64.78	3.77	10.54	4.21	12.28	4.42
Day type	**1705**						
Weekdays	1297 (76.07)	64.09	4.24	11.71	3.98	11.52	4.46
Weekends	408 (23.93)	70.77	0.15	1.91	3.86	17.99	5.32
Periods	**1705**						
Regular	1268 (74.37)	64.91	4.24	10.39	3.97	11.97	4.52
Holiday	437 (25.63)	67.97	0.43	6.39	3.89	16.25	5.07
Feeling ill	**1700**						
Yes	45 (2.65)	86.39	0.83	0.28	1.85	8.42	2.23
No	1655 (97.35)	65.21	3.34	9.64	4.02	13.18	4.61
Family status	**1008**						
Living with children	238 (23.61)	64.63	0.49	15.30	3.66	11.15	4.77
Living without children	770 (76.39)	61.44	0.17	14.53	4.35	14.21	5.30

#### Uncertainty

We applied a stratified bootstrap of the participant data (including social contact and time use data) by 5-year age categories to construct confidence intervals for the outcome. We created 1000 bootstrap replicates for each imputed dataset ($\mathcal {M}$=10), resulting in 10,000 datasets [26] (see Additional file 1 for more details).

#### Participant weights

Participant contributions to mixing patterns were weighted to account for sampling probabilities based on the joint distribution of age, household size and day type: holiday/regular periods and weekday/weekend. Census data for Belgium from 2011 (http://epp.eurostat.ec.europa.eu) was used as reference and weights were constrained to a maximum of 3 to reduce the impact of the corresponding observations.

#### Time use patterns

We explored time use patterns by calculating the proportion of time at each location as reported by participants. To limit data sparseness, we combined “kinder-garden” with “school”, and also “family” and “leisure” with “other”. Reported proportions were analysed in relationship with age, gender, weekday/weekend, period (regular/holiday), health state (ill or not ill) and family status. The latter was only defined for adults between 25 and 65 years of age as living with or without children less than 13 years old. We used 7 age categories in line with the Belgian education and employment system; 0–2 years, 3–5 years, 6–11 years, 12–17 years, 18–44 years, 45–64 years and 65–90 years of age. We also performed an additional analysis of the reported locations using 5-year age groups to study gender-specific differences in time use (refer to  Additional file 2: Fig. S2).

The proportion of time spent at different locations during one day can be analysed as compositional data. This is a special type of multivariate data, with each proportion being non-negative and the total sum adding up to one. A divergence based regression modelling technique is used to explore time use at each location [27]. More specifically, we used Kullback-Leibler divergence to minimize the distance between the observed and the fitted compositions with respect to the coefficients of the regression (Additional file 3). We used the 10,000 datasets generated through MICE and bootstrapping to construct weighted confidence intervals for all parameter estimates [28].

#### Contact patterns taking into account time use and distance

We used a GAMLSS model [29] for analysing the determinants of social contact patterns, taking into account the time spent at each location as explanatory variables. To handle classification issues in this sub-analysis, we aggregated all location types into “home”, “work”, “school” and “other”.

We linked social contact and time use data to obtain for each social contact the time spent at the reported location and the distance from home. Participants were able to report multiple distances for one location type over different time-slots in the time use part. For example, one could report “other” at 2 km and 10 km from home, for shopping in the morning and sport activities in the evening. As such, we applied a probabilistic link procedure between a social contact and the distance from home, based on the relative time spent at each distance per location type. The interaction between travel and social contact patterns has been studied unconditionally and conditionally upon presence at each distance, i.e., the former presents the population average and the latter is in line with the individual-level perspective. To elaborate on social contact dispersal, we classified participants by age using a cutoff of 18 years (child and adult), gender and type of day (regular weekday, weekend, holiday). We excluded the contacts with missing distance (±7%) to calculate the weighted average number of contacts by distance and by age, gender and day type.

#### Social contact matrices and R$_{0}$

The matrix **M**$^{dt}$, representing the mean number of contacts at distance *d* during one day of type *t*, can be estimated by the following expression:

1 $$\begin{aligned} {m}_{ij}^{dt} = \frac{\sum _{p=1}^{P_i}w_p y_{ijp}^{dt}}{\sum _{p=1}^{P_i}w_p}, \end{aligned}$$

where $P_i$ is the number of participants in age class *i*, $w_p$ the weight for participant *p* and $y_{ijp}^{dt}$ the reported number of contacts made by participant *p* of age class *i* with someone of age class *j* at distance *d* during one day of type *t*. The social contact matrix $c_{ij}^{dt}$, representing the per capita daily contact rate between age classes by distance and type of day, was calculated as

2 $$\begin{aligned} c_{ij}^{dt} = \frac{m_{ij}^{dt}}{N_j} \end{aligned}$$

with $N_j$ the population size in age class *j*, obtained from census data.

The next generation matrix *G* with elements $g_{ij}$ indicates the average number of secondary infections in age class *i* through the introduction of a single infectious individual of age class *j* into a fully susceptible population [30]. Assuming a rectangular population age-distribution, the next generation matrix at distance *d* during one day of type *t* is defined by:

3 $$\begin{aligned} G^{dt} = \frac{ND}{L} C^{dt} q, \end{aligned}$$

with *N* the population size, *D* the mean duration of infectiousness, *L* the life expectancy, $C^{dt}$ the contact matrix at distance *d* during one day of type *t* and *q* the proportionality factor. The basic reproduction number R$_{0}$ can be calculated as the dominant eigenvalue of the next generation matrix. To compare transmission dynamics by distance, we calibrated the parameters of Eq. (3) so that, without loss of generality, based on the population-based social contact matrix for a regular weekday, $R_0$ for regular weekdays using all reported contacts equals 2, averaged over all bootstrapped and imputed datasets.

#### Exposure time matrices

We calculated the age-specific exposure times based on time use data following the Proportionate Mixing Assumption (PTM) as previously used by [10]. As such, for one single location and time slot of the survey day, we calculated the exposure time of a participant to the other participants proportionally to their relative participation in that location. We used 17 categories; 0–2 years, 3–5 years, 6–11 years, 12–17 years, 18–25 years, 5-year age categories between 25 and 80 years of age, and a closing category of 80–90 years of age. Participants had to report their household members in terms of age, gender and whether they were present at home during the survey day. This allowed us to compute the time of exposure between members of the same household, which is formalized in the matrix **H**.

For locations other than home, the exposure time between people in age group *i* and *j* at specific location *l* and time slot *s*, $t_{ij}^{ls}$, can be computed, under the PTM assumption, as follows:

4 $$\begin{aligned} t_{ij}^{ls}=k_{i}^{ls} \; \;\frac{k_{j}^{ls}}{k_{.}^{ls}} \; d^{s}, \end{aligned}$$

where $k^{ls}_{i}$ and $k^{ls}_{j}$ are the number of people in age group *i* and *j* present at location *l* during time slot *s*, respectively, $k_{.}^{ls}$ is the sum over all age classes at location *l* during time slot *s* and $d^{s}$ is the duration of each time slot *s*, in hours. From (4), we can compute the time of exposure between people in age group *i* and in age group *j*, referred to as matrix **T**, as follows:

5 $$\begin{aligned} t_{ij}=\sum _{s=1}^{S}\sum _{l=1}^{L}k_{i}^{ls} \; \;\frac{k_{j}^{ls}}{k_{.}^{ls}} \; d^{s}, \end{aligned}$$

The sum of **T** and **H** determines the overall exposure time matrix **E**, with elements $e_{ij}$:

6 $$\begin{aligned} e_{ij}=t_{ij} + h_{ij}. \end{aligned}$$

The response matrix **E** contains non-negative quantities that are considered to follow a mixed discrete-continuous distribution, comprising value Y = 0 with probability $p_{0}$ and a value $Y = Y_1$ $\in (0,\infty )$ with probability (1- $p_0$). The R package gamlss.inf [31] was used to model **E**. For the discrete part (zero or not), we created a binary response variable to fit a binary logistic model. For the continuous part ($Y>0$), different distributions, including Gamma, Inverse Gaussian, Inverse Gamma, Log-normal, Weibull and Pareto were tested. Participants weights were taken into account and model selection was based on the Akaike information criterion (AIC).

#### Suitable contact matrices

The “suitable contact” approach assumes that not all social contacts are effective for disease transmission and that long duration and more intimate contacts are more likely to be relevant. To construct suitable contact matrices, we followed the procedure of De Cao et al. [11], which considered contacts and exposure between age classes *i* and *j* ($c_{ij}$ and $e_{ij}$, respectively). Let $u_{ij}$ be a random variable representing the number of suitable contacts between age group *i* and *j*. Then the expected number of suitable contacts is given by the product of the average number of contacts $c_{ij}$ and the proportion of these contacts that are suitable for transmission $1-\exp (-e_{ij}/c_{ij})$ where $\exp (-e_{ij}/c_{ij})$ is the Poisson probability that a contact is not suitable. Therefore the probability of infection $\beta _{i,j}$ is given by

7 $$\begin{aligned} \beta _{ij} =q_{1} \, u_{ij}, \end{aligned}$$

where $q_{1}$ is a constant disease-specific transmission coefficient and

8 $$\begin{aligned} u_{ij}=c_{ij} \; (1-\exp (-q_{2} \; e_{ij}/c_{ij})), \end{aligned}$$

with $q_{2}$ as the fraction of total exposure time between age groups that is suitable for transmission.

#### Fitting mixing matrices to parvovirus-B19 and VZV serological data

The social contact and the time use approach rely on the assumption that the number of potentially infectious contacts between people in different age categories is proportional to their total number of social contacts and time of exposure, respectively. Using our estimated age-specific social contact and exposure time matrices to estimate the parameters ($q_{1}, q_{2}$) from Eqs. 7 and 8 to capture the model-based disease prevalence under endemic equilibrium. As such, we identified values for these parameters that maximize the likelihood to obtain the observed prevalence of parvovirus-B19 and VZV. We followed the methods described in [32] and used a compartmental model with a mixing matrix between different ages including Maternally-derived immunity, Susceptible, Infectious and Recovered infection states (MSIR) to account for the disease dynamics of VZV [3, 11, 32]. For parvovirus-B19, we used an MSIRWb model [32], which is an MSIR model augmented with waning immunity and boosting in the Recovered compartment. In both models, and without loss of generality, we assumed newborns to be fully protected by maternal antibodies until 6 months of age [32], after which they become susceptible.

#### Fitting mixing matrices to influenza-like illnesses (ILI) incidence data

A dynamic, differential-equation, age-structured, Susceptible-Exposed-Infectious-Recovered (SEIR) model was used to fit different mixing matrices to ILI incidence data from 2010–2011. The weekly ILI incidence was recorded by the general practitioners (GPs) network, which covered 1.75% of the population in Belgium [22, 23]. Because we only considered one season (52 weeks from week 40 in 2010 to week 39 in 2011), demography is not included in the model (see Additional file 5 for more details of the model description and parameters). We used an average latent period of 1 day and an average infectious period of 3.8 days based on the literature [33, 34]. We estimated the model parameters by minimizing the sum of squared differences between the observed ILI incidence rate and the predicted incidence rates. The latter was performed with the “optim” function in R based on the “L-BFGS-B” method of Byrd et al. [35], which allows box constraints and uses a limited-memory modification of the BFGS quasi-Newton method. The following parameters were estimated: age group-dependent disease-specific proportionality factors $q_{i}$ (also based on the social contact hypothesis [2]) and a scaling factor. The scaling factor is used to calibrate the predicted incidence rates to the observed incidence rates, which accounts for those individuals with influenza who do not seek medical attention (the consultation rate), and which might absorb incorrect model assumptions or parameter mis-specification [33, 34, 36].

## Results

### Time use patterns

The final sample size for the analysis is 1707 participants, as reported in [18], though 49 participants left the time use part entirely blank. People older than 45 years of age accounted for the highest proportion of missing data for at least one time slot (Table 1). People spent on average about two thirds of their day at home.

The elderly over 65 years of age and children less than 6 years old reported the highest proportion of time spent at home. Time at work is observed to be higher for males than for females, the opposite is observed for time spent at home. Temporal factors such as day of the week and holiday periods had a large impact on time use.

Health status was also linked to substantial changes in participants’ time use. Participants did report an increase of 5 h at home when feeling ill and almost no time at work. However, the time spent at other locations is still substantial when people reported feeling ill. Adults between 25 to 65 years of age living with children under 13 years of age reported more time spent at home compared to those without children.

We analysed the reported locations by gender more in detail and observed that the average time at work is similar for males and females until the age of 30 years (Additional file 2: Fig. S1). From the age of 30 years onward, females reported on average less time at work compared to males in the same age group. We observed the highest differences in age groups [40,45), [50,55) and [55,60) in which males reported 10% more time at work compared to females. The reported time at work declined after 65 years of age for both genders.

The reported time for transport was similar for males and females. Time spent at “other” places was similar between males and females in general, except for the age categories [6,12) and [18,25) in which males reported 6% more time spent at “other” locations compared to females.

We analysed the time use patterns with a divergence-based regression model with multinomial logit link function including gender, age, day type and period. Table 2 shows all parameter estimates and 95% confidence intervals. After controlling for age and temporal factors, time at work reported by males is significantly higher compared to females. In reverse, females reported more time at home than males. The gender-specific difference in time spent at school was not statistically significant. Age had a significant effect on presence at home, school and work but not on the reported time at location category “other”. Temporal factors played a crucial role in the time use patterns, particularly on the presence at work and school. In addition, we observed a clear increase in the reported time in the “other” category during regular weekdays vs. weekends. This denotes compensation behavior of people not at work or at school, which needs to be considered when modeling weekend days.

**Table 2:** Parameter estimates and 95% confidence intervals of the divergence-based regression analyses for location-specific time use patterns

Variable	Category	Home	School	Work	Other places
Intercept		2.72 [2.40;3.10]*	1.62 [1.22;2.03]*	− 1.40 [− 9.16;-0.37]*	1.18 [0.80;1.55]*
Gender	Male	− 0.17 [− 0.35;− 0.03]*	− 0.14 [− 0.41;0.10]	0.37 [0.13;0.57]*	− 0.19 [− 0.4;-0.01]*
Ref.: Female					
Age	[0-3) years	1.05 [0.55;1.64]*	0.44 [− 0.23;1.14]	− 7.35 [− 51.28;-2.61]*	0.30 [− 0.29;0.97]
*Ref. : [12-18) years*	[3-6) years	0.55 [0.08;1.13]*	0.44 [− 0.08;1.09]	− 7.38 [− 40.00;-0.77]*	− 0.17 [− 0.70;0.46]
	[6-12) years	0.52 [0.05;1.03]*	0.74 [0.24;1.29]*	− 7.5 6 [− 44.18;− 1.99]*	0.34 [− 0.12;0.89]
	[18-25) years	− 0.04 [− 0.5;0.42]	− 0.53 [− 1.10;-0.01]*	2.20 [1.09;9.82]*	0.06 [− 0.43;0.56]
	[25-45) years	0.01 [− 0.36;0.37]	− 3.74 [− 4.53;− 3.09]*	2.94 [1.93;10.71]*	− 0.13 [-0.51;0.27]
	[45-65) years	0.09 [− 0.31;0.44]	− 4.17 [− 5.14;− 3.48]*	2.55 [1.52;10.24]*	− 0.04 [− 0.42;0.35]
	65+ years	0.17 [− 0.22;0.58]	− 7.81 [− 22.25;-4.49]*	− 0.93 [− 2.57;6.46]*	**− **0.35 [− 0.74;0.09]
Temporal factor	Holiday weekday	0.4E−1 [− 0.23;0.23]	− 2.48 [− 3.52;− 1.84]*	− 0.24 [− 0.56;0.07]	0.26 [0.02;0.53]*
Ref. : Regular weekday	Regular weekend	0.11 [− 0.09;0.35]	− 3.36 [− 5.15;− 2.55]*	− 1.92 [− 2.56;− 1.42]*	0.54 [0.29;0.81]*
	Holiday weekend	0.23 [− 0.09;0.55]	− 5.34 [− 19.6;− 4.09]*	− 1.90 [− 2.69;− 1.32]*	0.60 [0.27;0.94]*

The asterisks (*) denote the confidence intervals which do not include zero

#### Social contacts patterns taking into account time use

We analysed the number of reported social contacts with a zero-inflated negative binomial model and observed that the overall number of contacts was inversely associated with the time spent at home, and positively associated with the time spent at school or work (Table 3). The number of contacts at school or work tends to increase with the time spent in these settings. We observed a gender effect, implying that males tend to have on average fewer contacts compared to females per time-unit. However, there was no statistical difference in the number of contacts at different locations between males and females. With respect to age, the highest overall number of contacts were observed among children up to 18 years of age and among people in the working age (25–65 years of age). The age effect was not observed for the social contacts at home and school, but observed for the social contacts at other places.

**Table 3:** Parameter estimates and p-values of zero-inflated negative binomial models for the total number and location-specific social contacts using age, gender, temporal and time use ﻿covariates

Variable	Category	Overall	Home	School	Work	Other places
Intercept		3.14(0.20)	− 0.46(0.16)	1.25(1.32)	0.96(0.36)	3.93(0.25)
Time use (in hours)						
Home		− 0.07(0.05E−1)**	0.04(0.04E−1)**	0.04(0.05)	− 0.05(0.01)**	− 0.15(0.01)**
School^a^		0.06(0.01)**	0.01(0.01)	0.14(0.05)*	–	− 0.10(0.02)**
Work^b^		0.08(0.01)**	− 0.01(0.06E−1)*	–	0.43(0.02)**	− 0.16(0.01)**
Gender	Male	− 0.11(0.03)**	− 0.05E−1(0.03)	0.01(0.19)	-0.11(0.09)	− 0.05(0.05)
*Ref.: Female*						
Age group	[3-6) yrs	0.47(0.19)*	0.05(0.17)	0.50(0.51)		0.41(0.20)*
*Ref.: [0-3) yrs*	[6-12) yrs	0.41(0.18)*	0.09(0.13)	0.49(0.54)		0.51(0.18)**
	[12-18) yrs	0.33(0.18)	0.09(0.14)	0.38(0.64)		0.50(0.18)**
	[18-25) yrs	0.08(0.18)	0.05(0.15)	–		0.18(0.17)
	[25-45) yrs	0.36(0.17)*	0.09(0.13)	–	–	0.49(0.16)**
	[45-65) yrs	0.33(0.17)*	0.20(0.13)	–	–	0.47(0.16)**
	65+ yrs	0.05(0.18)	0.08(0.13)	–	–	0.42(0.17)*
Household size		0.08(0.01)**	0.28(0.01)**	− 0.18(0.09)	− 0.05(0.04)	0.06(0.02)**
Class size		–	–	0.02(0.02)		
Temporal factors	Holiday weekday	− 0.05(0.04)	0.03(0.04)	− 5.83(1.21)**	− 0.46(0.13)**	0.08(0.07)
*Ref.: Regular weekday*	Regular weekend	0.11(0.05)*	0.02(0.05)	− 3.74(0.26)**	− 0.48(0.11)**	0.33(0.08)**
	Holiday weekend	− 0.11(0.07)	− 0.02(0.05)	− 37.77(21.31)	− 1.21(0.18)**	0.13(0.10)
Sample size		**1707**	**1707**	**227**	**1008**	**1707**

*p < 0.05; **p < 0.01; $^{a}$ only include participants that spent time at school and reported class size; $^{b}$ only include participants from 25 to 65 year old; – not applicable

Household size was positively associated with the total number of contacts, contacts at home and other places, however household size showed no significant effect on the social contacts at school and at work. Class sizes did not have a clear effect on the number of school contacts. As expected, we observed an explicit link between temporal factors and the number of school and work contacts.

#### Social contact dispersal

**Fig. 2:**
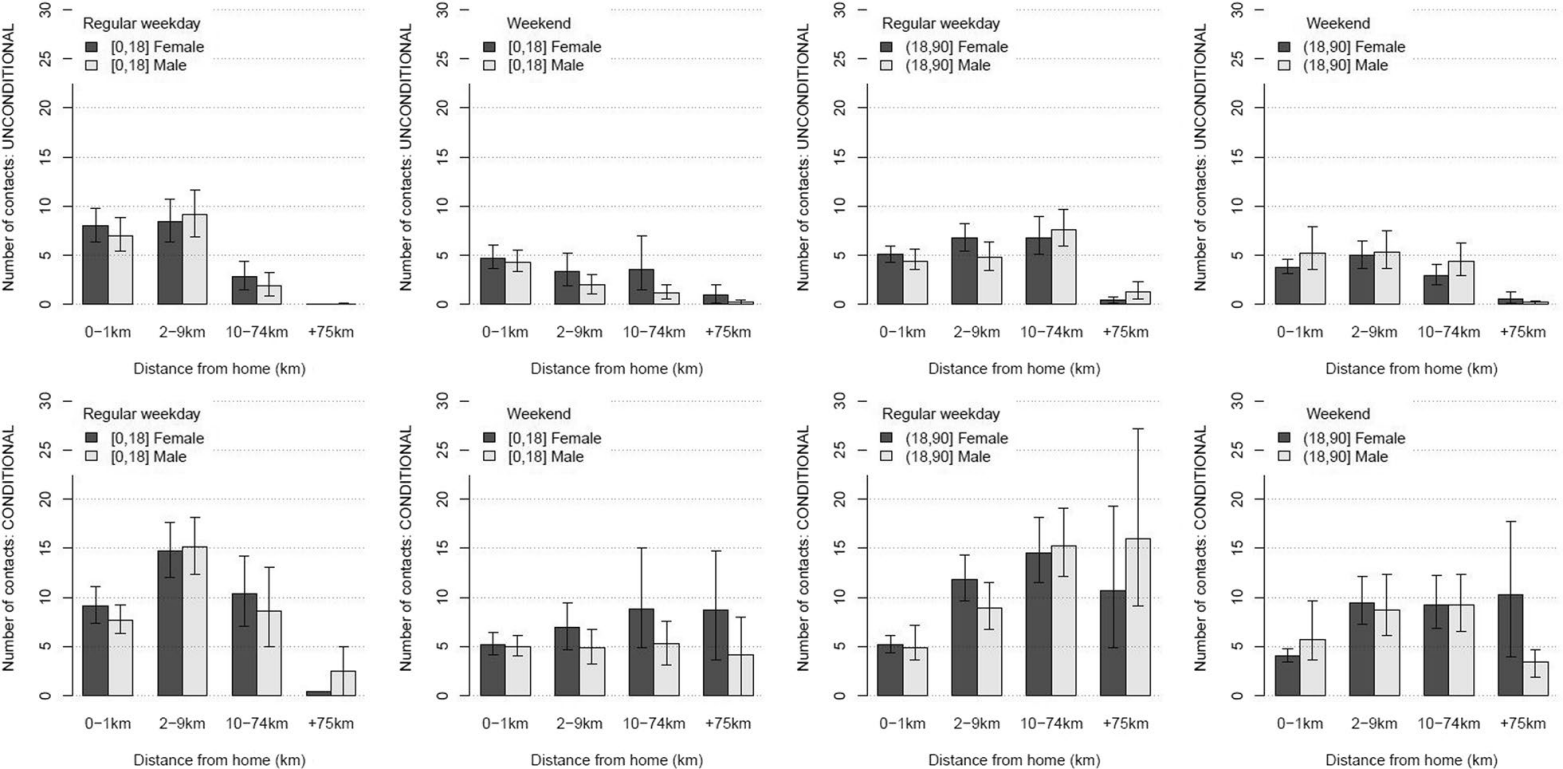
Number of contacts by distance. Number of contacts by distance unconditional (top) and conditional (bottom) upon presence by age (left/right) and gender (color) during regular weekdays and weekends. The median is represented by the bars, 95% CI by the whiskers

To explore social contact dispersal, we analyzed the social contact data by distance in combination with age, gender and type of day (Fig. 2). At population level, i.e. unconditional upon whether or not people travel, we observed most contacts for children (0–18 years of age) in the category 0–9 km from home during weekdays and very few contacts beyond 10 km from home. This pattern was the same for boys or girls. During weekends, children reported a decline in the number of contacts by distance, but less so for girls. For adults (18+ years of age), we observed an increase by distance on weekdays up to 10–75 km from home. On average, people reported almost no contacts 75+ km from home. Females reported more contacts up to 9 km from home compared to males, though the opposite holds for the category 10–74 km. For weekends, we observed a decline in the number of contacts beyond 10 km from home, both for males and females.

The conditional results, i.e. looking at the number of contacts upon having traveled a certain distance, showed a different result compared to the unconditional (or population based) results. For children, the conditional results for weekdays show a clear maximum in the category 2–9 km from home. The number of contacts at 10–74 km from home equals the number of contacts close to home. During weekends, the reported number of contacts for girls increased with distance though the social contact behavior for boys seemed indifferent with distance up to 74 km. For adults, the number of social contacts during weekdays at 2–9 km and 10–74 km from home was two and three times the number of reported contacts at home, respectively. Also for the last distance category (75+ km from home), we observed an increase compared to the contacts at home but with large uncertainty. If adults leave home during weekends, the reported number of contacts by adults seems indifferent to the distance up to 74 km. Only at 75+ km from home, females reported fewer social contacts, compared to males. In conclusion, the number of contacts decreased by distance at population level, though if people made the effort to go somewhere, they made it count in terms of their social contact behavior.

#### Transmission dispersal and R$_{0}$

**Fig. 3:**
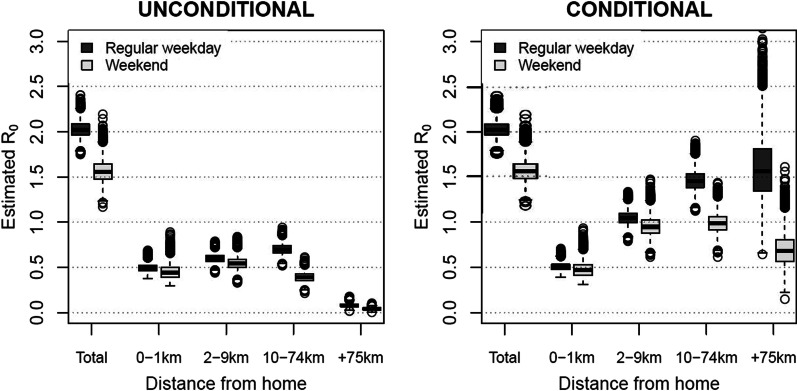
Estimated basic reproduction number R$_{0}$ by distance. R$_{0}$ by distance, calculated as the leading eigenvector of the distance-specific social contact matrix and calibrated so the median R$_{0}$ for regular weekdays equals 2 based on population-based (unconditional) contact matrices. For each distance category, we used the unconditional and conditional social contact patterns. The median is represented by the horizontal line in the box (75% interval), the whiskers denote the 95% confidence intervals and the dots are outliers

The social contact dispersal as illustrated in Fig. 2 induces distinct transmission potential by distance. We calculated social contact matrices by distance for child/adult interactions for each bootstrapped dataset (e.g.,  Additional file 4: Figs. S1 and S2) and corresponding R$_{0}$ values. We calibrated the disease-specific proportionally factor *q* for a flu-like disease with median R$_{0}$ = 2 based on the population-based social contact matrix for a regular weekday. The estimated R$_{0}$ values by distance should be interpreted as the relative transmission potential and are presented in Fig. 3. On the population level, the transmission potential slightly increased until 74 km from home during regular weekdays. This can be explained by the strong assortative mixing 2–9 km from home and the work-related mixing between adults 10–74 km from home (Additional file 4: Fig. S1). The overall reduction in the transmission potential during weekends, can be explained by the fewer number of contacts for the distance category 10–74 km from home. Social contacts at 75+ km from home had almost no impact on the unconditional transmission potential.

The transmission potential conditional upon presence showed a clear increase with distance from home during regular weekdays (Fig. 3). This effect was similar for weekends, however with a decrease of the estimated R$_{0}$ for the last distance category. In general, the estimated transmission potential increased by distance from an individual-based perspective.

#### Exposure time and social contact matrices

**Fig. 4:**
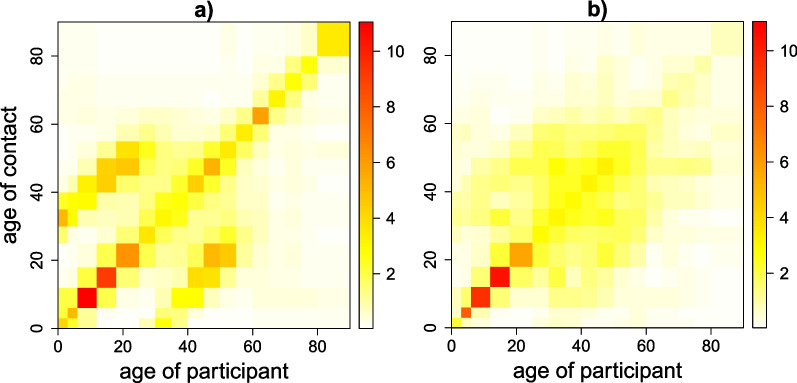
Age-specific mixing pattern. Mixing patterns by age represented by weighted exposure time in hours (**a**) and weighted average number of contacts (**b**)

We used a zero-adjusted log-normal model on the time use data to estimate the age-specific exposure matrices. The resulting exposure matrix reflected contributions from exposure at home, at school, at work, and at other locations (Fig. 4). The main diagonal indicates that people tend to spend time with people of similar age. The two sub-diagonals represent the mixing pattern between generations. We also calculated the corresponding social contact matrix representing the weighted average number of contacts by age. Both matrices showed strong assortative mixing by age, especially for young children and teenagers. Especially the exposure matrix showed strong interaction among family members, as a result of the reported time at home. This pattern is very clear in the location-specific exposure matrix at home (Additional file 3: Fig. S1).

**Fig. 5:**
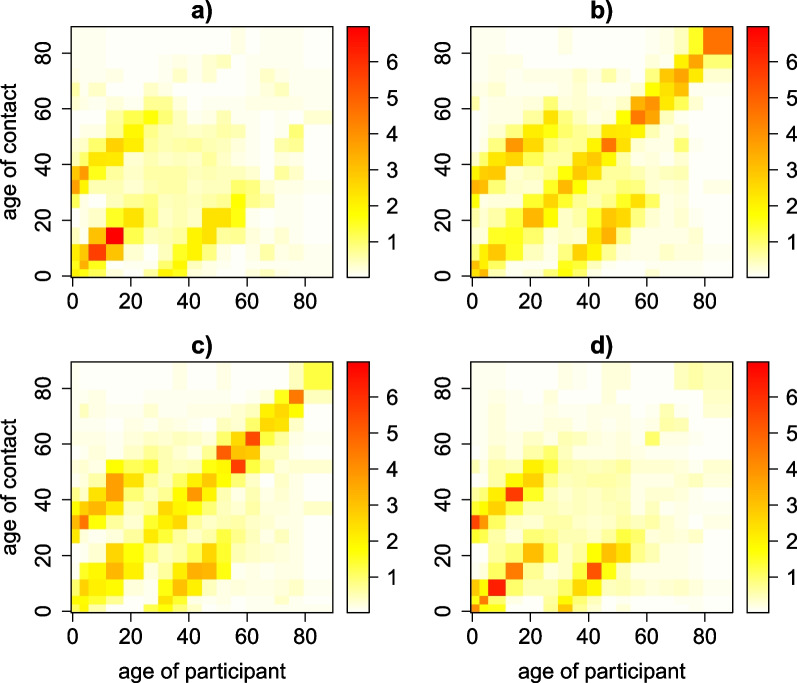
Estimated age- and gender-specific exposure time. male–male (**a**), male–female (**b**), female–male (**c**) and female–female (**d**). The color scale indicates the exposure time in hours from low (white) to high (red)

Time reported at home constituted on average up to ±66% of the total time per day, while contacts at home only accounted for ±18% of the total number of contacts. In contrast, the impact of employment is higher for the contact matrix compared to the exposure matrix (±40% of the total number of contacts though only ±9% of total time per day). Gender- and age-specific exposure time matrices are shown in Fig. 5. We observed more pronounced assortative mixing pattern in the same-gender matrices for children and teenagers, though more different-gender exposure time for adults older than 50 years of age. In addition, the interaction between mothers and daughters seemed more pronounced compared to other child-adult interactions.

#### Fitting social contact and exposure matrices to parvovirus-B19 and VZV serological data

We tested the value of exposure and social contact matrices as a proxy for effective contacts governing transmission by their value in MSIRWb and MSIR models for parvovirus-B19 and VZV, respectively. Model estimations were compared to serological data and model selection was based on the AIC criterion. If we used the overall social contact matrix regardless of intimacy, location and duration, the “time use approach” implied a better fit for both parvovirus-B19 and VZV (Additional file 5: Tables S2 and S3). Close contacts lasting more than 4 h, provided the best proxy for the transmission dynamics of parvovirus-B19. For VZV, the social contact matrix based on close contacts of at least 1 h did improve the contact approach, though the AIC was still higher compared to the time use approach.

**Fig. 6:**
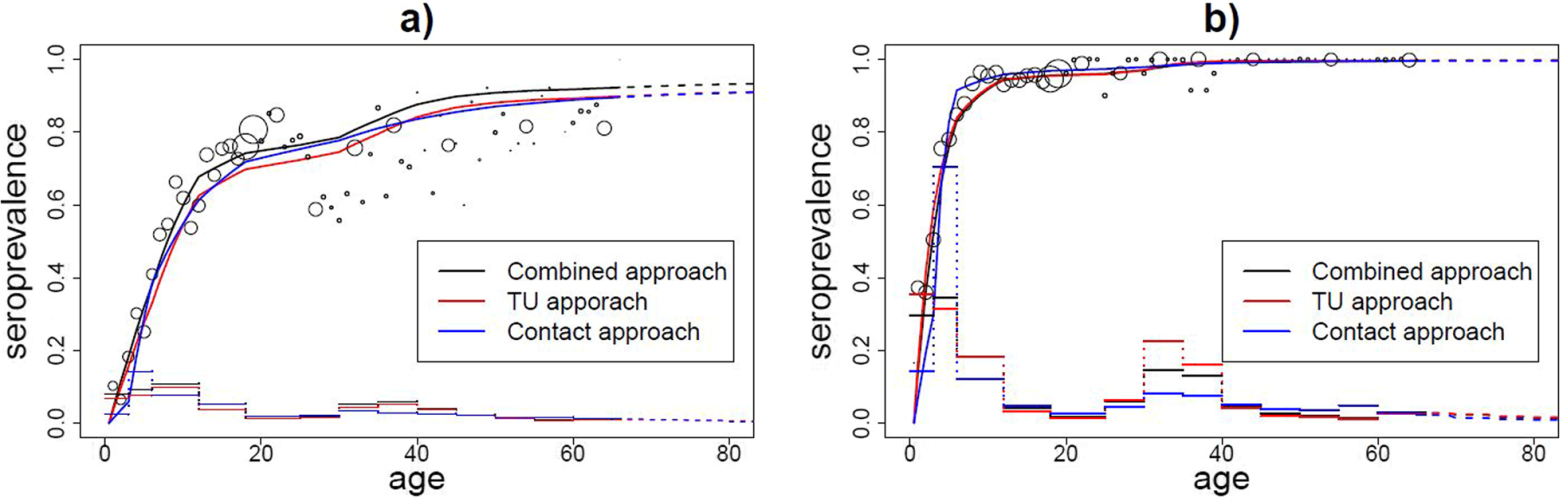
The fitting results to serological data. The fit of 3 different matrices to serological parvovirus-B19 (**a**) and VZV (**b**) data for Belgium

**Fig. 7:**
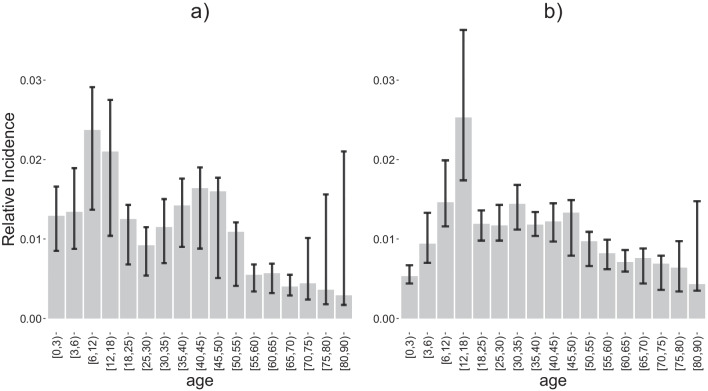
Relative incidence by age groups. Time use data (**a**) and social contact data (**b**)

The suitable contact approach, which accounts for both the number of social contacts and time of exposure, scored slightly better for parvovirus-B19 compared to the contact approach, but not in comparison to the time use approach. For VZV, the application of suitable contact estimates provided the overall best fit with the lowest AIC. The estimated proportionality factor $q_{2}$ of exposure data with respect to the overall model prediction is much higher for VZV (0.94) than for parvovirus-B19 (0.13), which implied a higher number of suitable contacts for transmission of VZV. Our best-fitting models estimated a reproduction number of 1.9 [1.7; 2.1] and 7.8 [6.8; 8.5] for parvovirus-B19 and VZV, respectively. The estimated prevalence and force of infection by the 3 approaches were quite similar (Fig. 6). We did observe differences in the predicted age-specific relative incidence by the time use and social contact approaches (Fig. 7). The highest relative incidence obtained by the social contact approach was among children in the age group [12,18), while the highest relative incidence obtained by the time use approach is among people in age group [6–12). The latter also predicts a relatively higher incidence in adults between 35 and 50 years of age.

#### Fitting social contact and exposure time matrices to ILI incidence data

Social contact and exposure time matrices were used to compute transmission rates in the dynamic, differential-equation SEIR model for ILI incidence data. The model comparison was based on the least square score, a direct measure of goodness of fit with smaller values indicating a better fit to the ILI incidence data. The intimacy and duration of contacts seemed to matter for the ILI transmission modelling: physical contacts provided a better proxy of transmission than non-physical contacts, while contacts lasting at least 1 h provided the best fit, compared to contacts of other duration. The use of exposure time matrices provided a better fit for ILI, compared to the use of overall contact matrices (Additional file 5: Table S4, Additional file 6). $R_{0}$ estimated from the best model is 1.43 and scaling factor is 0.266. The integration of contact matrices and exposure time matrices did not improve the fit. Additional file 5: Figure S1 shows the fit of different matrices to ILI incidence rate. The estimated incidence rates from models using suitable contact matrices and exposure time matrices are almost overlapping, with only a slight difference at the seasonal peaks of transmission.

**Fig. 8:**
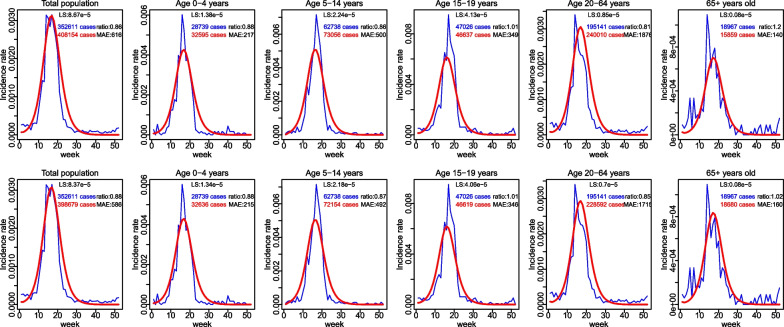
The fitting results to ILI incidence data. Observed ILI incidence rates in Belgium 2010–2011 (blue) and corresponding model-based estimates (red) using overall contact matrices (top row) or exposure time matrices (bottom row)

Figure 8 shows the fit of the models that used overall contact matrices and exposure time matrices, for the total population and age group-specific populations. For each case, we present the least square value, the observed number and modeled based estimates of ILI cases, their ratio, and the mean absolute error (MAE). The uncertainty intervals around the fitted model estimates were very large, so we did not include them in the figure. For the total population, the estimated ILI cases captures quite well the weekly observed number of ILI cases. Overall contact matrices and exposure time matrices provided quite similar results for the total population and most of age groups, except for the age group of 65 years and older. More precisely, the use of exposure time matrices captured the observed ILI curve for this age group better than the use of overall contact matrices. In general, the quality of the fit does not differ substantially between age groups. The models tend to overestimate the number of ILI cases for children and teenagers aged 0–19 years and adults aged 20–64.

## Discussion

We analysed time use and social contact data and compared their use as proxy for effective contacts governing disease transmission for disease transmitted through the respiratory route. Our dataset is unique since it provides both time use and social contact data from the same participants, avoiding possible differences due to sample biases. In our analysis we identified the main drivers in shaping everyday time use and linked this info to social contact patterns.

The reported time use patterns in Belgium are quite similar to the published patterns for Italy [37], but different from the results from Zimbabwe [38]. In Zimbabwe, the working-age participants and children less than 6 years old reported much less time at work and school respectively, compared with participants of the same age group in Belgium and Italy. We found that males spent on average $\pm 2$ hours more at work than females, which is in line with previous work [39, 40]. We also found that both males and females living with children spend more time at home than people living without children, which is consistent with what was found in [39]. The expected temporal patterns were observed, with more time spent at “other” locations during weekends and holiday periods. The time spent at home seemed not to be affected by the type of day. Our gender-specific analysis of the time use data indicated that participants were prone to spend more time with the same gender when they are young, and more time with the other gender when they are older. This result differs from the observed gender-specific contact rates as reported in [18], where the assortativity is reported to be higher for same-gender contacts.

Power law dispersal has been useful to model disease counts [13–15], though questions remained whether this also holds for social contact behavior, the driver of transmission dynamics [2]. Danon et al. [16] showed a relation between clustering and distance from home, with high clustering within two miles, dominated by home contacts, but the highest value of clustering occurring at a distance 50 miles or more from home. The authors hypothesize that this might be due to differences in the purpose behind contacts made at various distances.

In our study, we observed an increase in the number of contacts by distance during weekdays, until an age-specific distance-threshold. A large-scale study in Taiwan [41] reported that 52.7% of contacts took place at a distance less than 1 km from home, 29.2% at a distance 1–9 km from home, 14.6% at a distance 10–49 km from home. In our study, this pattern was clearly age-specific. Half of our reported contacts during regular weekdays for children between [0–18) years of age took place less than 1 km from home. During weekends, we observed more contacts at +10 km from home. In general, we observed that the average number of contacts decreased by distance, though if people made the effort to travel, they made it count in terms of social contact behavior. As such, social contact patterns conditional upon presence at each distance provided useful info to inform individual-level behavior. A study in China quantified the distances from home based on the latitude and longitude of each reported contact and observed an increase in assortative mixing when contacts were made further from home [42]. We observed similar patterns with the construction of conditional social contact matrices by distance. A study in the United Kingdom [43] requested for infants to report the maximum distance travelled from home, but, to date, did not report results thereof. Other survey designs (e.g., [38]) included the distance between home and work but did not report results related to this information, yet.

The age-specific social contact and time use pattern followed a similar trend of assortativeness, which stresses once more the tendency for people to have contacts with someone of similar age. In addition to that, strong mixing among generations (parent–child) was present in both the social contact and time use matrices. The inter-generation mixing is mostly observed at home, and was more pronounced in the exposure time matrix than in the contact matrix. With our unified survey, we can confirm the contrasting effect of the relative small number of social contacts at home compared to the large amount of time spent at home, as observed in  [10, 38].

Social contact matrices provide useful data to estimate disease transmission dynamics in terms of the transmission dynamics and relative incidence [1, 2]. As such, we estimated the R$_{0}$ for each distance conditional upon presence, and observed a clear increase by distance. This reflects an increasing transmission potential by distance from the individual-level perspective. The latter is of interest for individual-based and some meta-population models where individuals join other sub-populations at distance. Assuming a constant (or decreased) social mixing behavior by distance conditional upon presence might not be optimal. Some individual-based models handled this by the use of location-specific mixing patterns irrespective of distance from home [44].

We compared the value of different social contact features (duration, physical/non-physical, etc.) to inform transmission models for parvovirus-B19 and VZV. By scoring the model-based prevalence with Belgian serological data, we found that physical contacts provided the best proxy for both parvovirus-B19 and VZV. In terms of contact duration, the best model fit was obtained with physical contacts of long duration (more than 4 h) for parvovirus-B19 and (more than 1 h) for VZV. Goeyvaerts et al. [3] reported the best fit to VZV with physical contacts of at least 15 min; this result is also in line with the study of [4]. However, in the previous studies, not all combinations in terms of contact duration and physical/non-physical contacts were analysed, which explains the new “best estimate” in our study.

We also compared the results of the contact approach, the time use approach and the suitable contact approach in fitting serological data of VZV and parvovirus-B19. In the case of VZV, the suitable contact approach provided the best fit, while for parvovirus-B19, the time use approach gave the best fit. Our results are consistent with the findings of De Cao et al. [11], although we observed much higher parameter estimates for $q_2$ (0.13 vs 0.001 for parvovirus-B19 and 0.94 vs 0.37 for VZV), but the confidence intervals of these parameter estimates were overlapping.

We also tested the value of contact matrices and exposure time matrices in fitting the dynamic transmission model to weekly ILI incidence data in the season 2010–2011 in Belgium. Exposure time matrices provided a better fit to ILI than overall contact matrices and the integration of these two types of matrices did not improve the fit to the data. Within the social contact approach, physical contacts provided a better proxy for the risks of influenza transmission than non-physical contacts. We found that $R_{0}$ of the best model is 1.43, this result is consistent with a systematic review of estimates of $R_{0}$ for different types of influenza [45] and a study in UK [34] that also used contact matrices to fit to ILI incidence data. However, this value is much lower than the seasonal estimates of the reproduction number in [33], in which physical contact matrices from the Belgian POLYMOD data were used to fit to ILI data over multiple influenza season from 2003 to 2009. Fewer ILI cases were reported in the season 2010–2011 as compared with previous seasons, which partly explained the difference in $R_{0}$. In addition, note that small differences in model parametrization entail substantial differences between the estimates of $R_{0}$, which was also mentioned in [33].

In our study, we combined information from time use and social contact data to gather information on human mixing patterns. One of the main advantages with respect to previous work is that both sources of information came from the same survey. To keep participants’ burden as low as possible, time use information was collected with rather large time slots and participants were asked to fill in only one location for each time slot. However, the comparison with more refined time use surveys performed in Flanders [39, 40] confirms that we were able to well characterize exposure patterns at an aggregated level. Therefore, we expect that this limitation did not substantially affect our results. Under the Proportionate Mixing Assumption, age-specific exposure matrices could be biased for some locations, e.g. public transportation or ‘Other’ location. In this study, we assume that age specific-contact patterns and time use patterns did not change over 10 years, the gap (in years) between the collection of serology and social contact data. This assumption is partially supported by the work of Hoang et al on contact patterns over a time span of 5 years [18].

## Conclusions

In conclusion, our work emphasises the value of both and the complementary information provided by time use and social contact data for analysing behavior and informing disease transmission models. Both data sources share a common value of being a good proxy for the transmission route of the pathogen. Combining social contact and time use data can provide a slightly improved measure of risk events with respect to VZV. Furthermore, our analysis based on data from the same survey is in line with studies that merged information from different surveys [10, 11]. This indicates that complementing social contact with independent time use data is a viable choice for the analyses presented here.

## Supplementary Information

**Additional file 1. **Missing data exploration and the list of variables included in the imputation model.

**Additional file 2. **Location-specific time use by family status, gender and age.

**Additional file 3. **Time use patterns and the mean daily exposure time among age groups across locations.

**Additional file 4. **Number of contacts and temporal social contact matrices by distance.

**Additional file 5. **Fitting results of social contact and exposure matrices to parvovirus-B19 and VZV sero-logical data, and ILI incidence data.

**Additional file 6. **Reference list for additional files.

## Data Availability

The social contact datasets analysed during the current study are available on Zenodo: 10.5281/zenodo.4059825. Incidence data are available from the authors (niel.hens@uhasselt.be) upon reasonable request and with permission of the Scientific Institute of Public health, especially for the ILI data.The R code can be found in https://github.com/hoanghvt/Time-use.git.
