# Supplementary material for: Exploring human mixing patterns based on time use and social contact data and their implications for infectious disease transmission models
Source: BMC Infect Dis. 2022 Dec 19;22:954. doi: 10.1186/s12879-022-07917-y (PMC9764639; doi:10.1186/s12879-022-07917-y)
Supplement: Supplementary file 1 — Additional file 1. Missing data exploration and the list of variables included in the imputation model. [file 12879_2022_7917_MOESM1_ESM.pdf]

Additional file 1: Missing data exploration and the list of variables included in the imputation model.

Missing data

Table S1: Missing frequency in time use data

Nr of not-filledin	0	1	2	3	4	5	6	7	8	9	10	11	12	13	14	15	16	17
Nr of participants	1576	14	12	10	8	4	5	3	6	3	3	1	2	1	1	6	3	49

Table S2: Missing frequency by diary versions. Version 1 is for children less than 13, version 2 is for participants from 13 to 65 and version 3 for participants older than 65.

Nr of not-filledin	Version 1	Version 2	Version 3
0	283	1012	281
1+	13	90	28

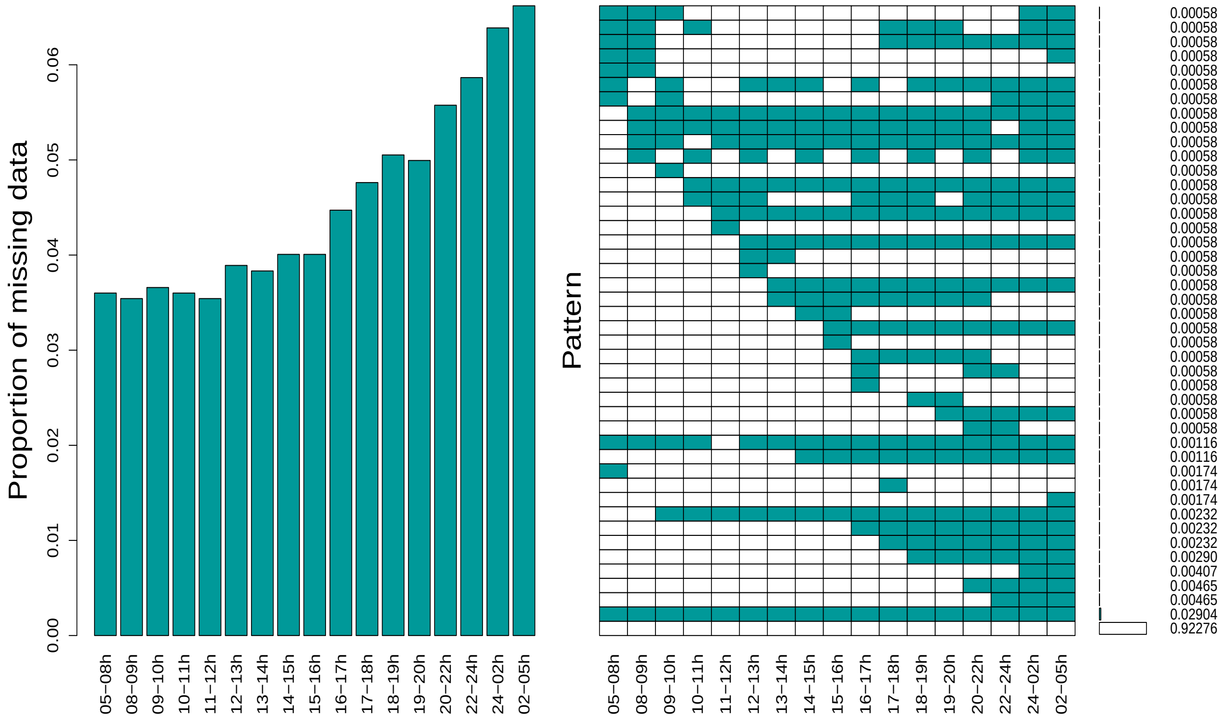

Figure S1: Missing data by time slots

Variables included in the imputation model

Table S3: Variables included in the imputation model

Nr	Variable name	Variable labels	Type	Value labels
1.	participant_age	Age of survey participants	Integer	
2.	participant_gender	Gender of survey participants	String 1 char	M: male<br>F: female
3.	dayofweek	Day of the week of filling in the diary	Integer	0: Sunday<br>1: Monday<br>2: Tuesday<br>3: Wednesday<br>4: Thursday<br>5: Friday<br>6 : Saturday
4.	holiday	Was the diary filled in during a public or school holiday?	String 1 char	Y: Yes<br>N: No
5.	time_use_location_1	Location where you spent the most time between: 5-8h	Integer	1: home<br>2: kinder-garden<br>3: school<br>4: workplace<br>5: transport<br>6: family<br>7: leisure<br>8: other<br>9: missing
6.	time_use_location_2	Location where you spent the most time between: 9-10h	Integer	As above
7.	time_use_location_3	Location where you spent the most time between: 10-11h	Integer	As above
8.	time_use_location_4	Location where you spent the most time between: 11-12h	Integer	As above
9.	time_use_location_5	Location where you spent the most time between: 12-13h	Integer	As above
10.	time_use_location_6	Location where you spent the most time between: 13-14h	Integer	As above
11.	time_use_location_7	Location where you spent the most time between: 14-15h	Integer	As above
12.	time_use_location_8	Location where you spent the most time between: 15-16h	Integer	As above
13.	time_use_location_9	Location where you spent the most time between: 16-17h	Integer	As above
14.	time_use_location_10	Location where you spent the most time between: 17-18h	Integer	As above
15.	time_use_location_11	Location where you spent the most time between: 18-19h	Integer	As above
16.	time_use_location_12	Location where you spent the most time between: 19-20h	Integer	As above
17.	time_use_location_13	Location where you spent the most time between: 20-22h	Integer	As above
18.	time_use_location_14	Location where you spent the most time between: 22-24h	Integer	As above
19.	time_use_location_15	Location where you spent the most time between: 24-02h	Integer	As above
20.	time_use_location_16	Location where you spent the most time between: 02-05h	Integer	As above
21.	time_use_location_17	Location where you spent the most time between: 05-08h	Integer	As above
22.	hh_size	Household size including participants	Integer	

Calculation of Confidence Interval (CI)

MI is used for the dataset $\mathcal{D} = \{\mathcal{D}^{obs}, \mathcal{D}^{mis}\}$ where the data matrix consist of both observed and missing values. For each of the $\mathcal{M}$ imputed datasets $\mathcal{D}_m$, $\mathcal{B}$ bootstrap samples are drawn which yields $\mathcal{M}$ x $\mathcal{B}$ datasets $\mathcal{D}^*_{m,b}$ where $b = 1, ..., \mathcal{B}$ and $m = 1, ..., \mathcal{M}$. In each of these datasets, the parameter of interest is estimated, $\hat{\theta}^*_{m,b}$. The pooled set of ordered estimates $\Theta^*_{MB} = \{\hat{\theta}^*_{m,b}; b = 1, .., \mathcal{B}; m = 1, ..., \mathcal{M}\}$ is used to construct the 1-2α % confidence interval for θ [4]:

$$[\hat{\theta}_{lower}; \hat{\theta}_{upper}] = [\hat{\theta}^{*,\alpha}_{MB}; \hat{\theta}^{*,1-\alpha}_{MB}] \tag{1}$$

Where $\hat{\theta}^{*,\alpha}_{MB}$ is the α percentile of the ordered estimates Θ^*_{MB}
