# Supplementary material for: Exploring human mixing patterns based on time use and social contact data and their implications for infectious disease transmission models
Source: BMC Infect Dis. 2022 Dec 19;22:954. doi: 10.1186/s12879-022-07917-y (PMC9764639; doi:10.1186/s12879-022-07917-y)
Supplement: Supplementary file 2 — Additional file 2. Location-specific time use by family status, gender and age. [file 12879_2022_7917_MOESM2_ESM.pdf]

Additional file 2: Location-specific time use by family status, gender and age

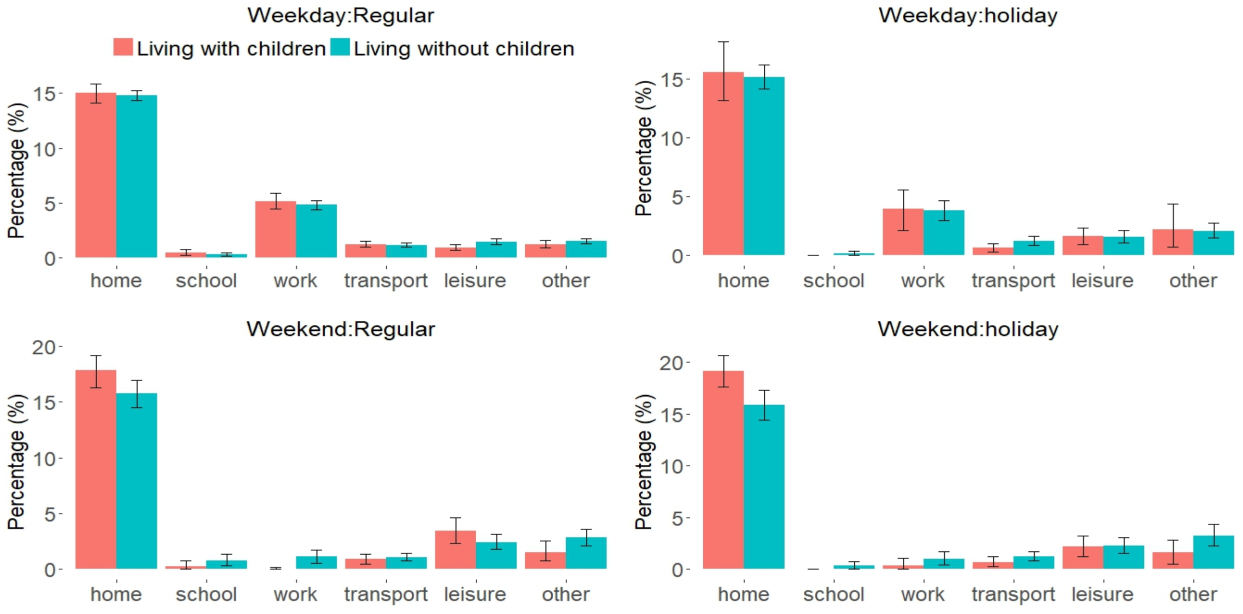

Figure S1: Time use per day by family status (population from 25 to 65 years of age)

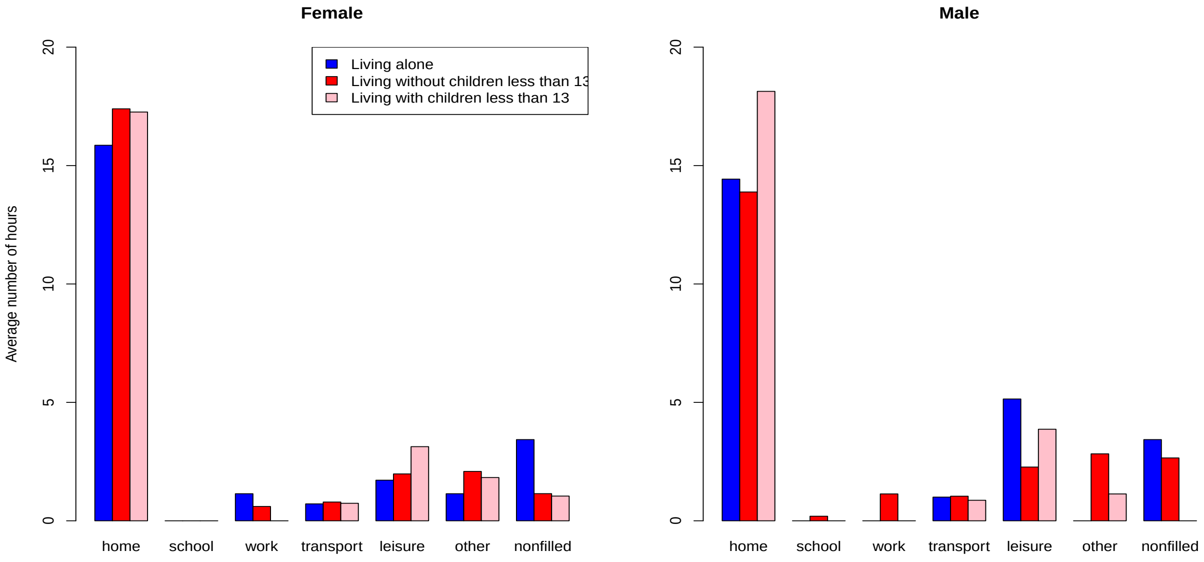

Figure S2: Time use per day by family status in holiday period (population from 25 to 65 years of age)

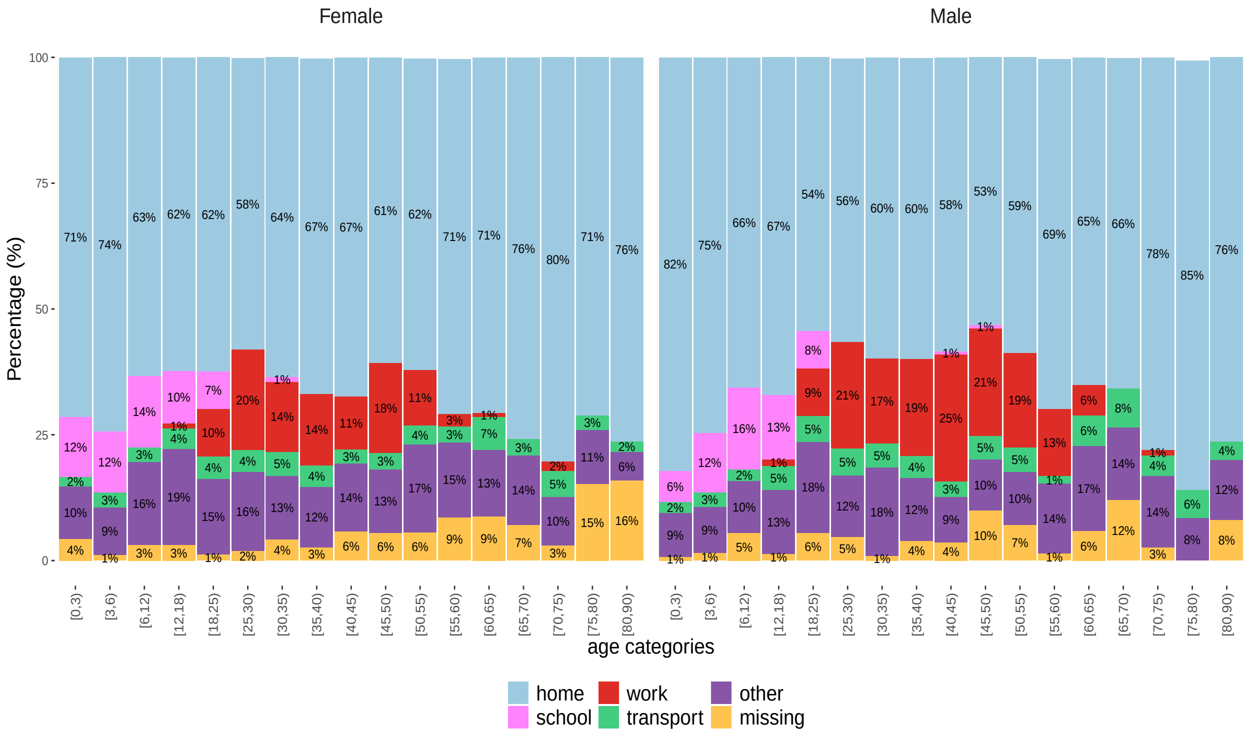

Figure S3: Time use per day over age categories of females and males (values below 1% are not shown)

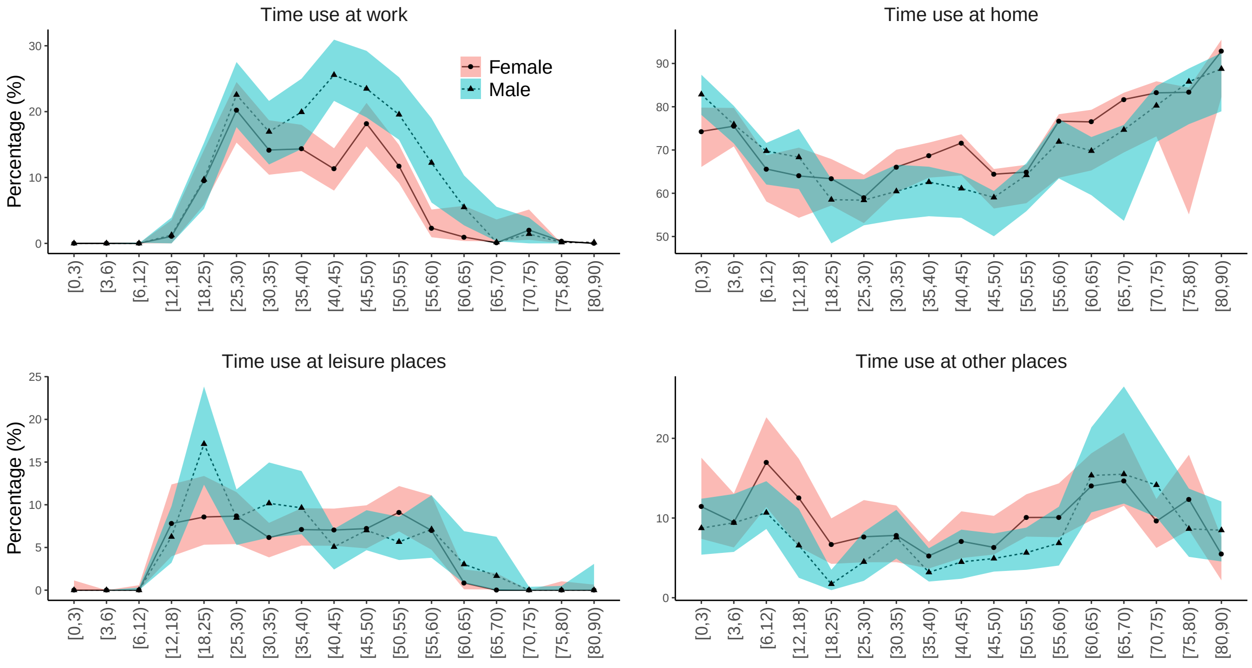

Figure S4: Time use of the male and the female over age groups
