# Supplementary material for: Exploring human mixing patterns based on time use and social contact data and their implications for infectious disease transmission models
Source: BMC Infect Dis. 2022 Dec 19;22:954. doi: 10.1186/s12879-022-07917-y (PMC9764639; doi:10.1186/s12879-022-07917-y)
Supplement: Supplementary file 3 — Additional file 3. Time use patterns and the mean daily exposure time among age groups across locations. [file 12879_2022_7917_MOESM3_ESM.pdf]

Additional file 3: Time use patterns and the mean daily exposure time among age groups across locations.

Kullback divergence method: This method is used to explore the determinants of time use pattern

The proportion of time spent at different locations during one day can be analysed as compositional data. The compositional data, a special type of multivariate data, is illustrated as below:

$$S^d = \left\{ (y_1, ..., y_D | y_i \geq= 0), \sum_{i=1}^{D} y_i = 1 \right\} (d = D - 1) \tag{2}$$

Kullback-Leibler divergence can express the dissimilarity between two probability distributions. In this case, it can be used for minimization of the distance between the observed and the fitted compositions with respect to the coefficients ([6]).

$$\sum_{j=1}^{n} \sum_{i=1}^{D} KL(y_{ij}, \hat{y}_{ij}) = \sum_{j=1}^{n} \sum_{i=1}^{D} KL(y_{ij}, f_{ij}\ (\beta; x)) = \sum_{j=1}^{n} \sum_{i=1}^{D} y_{ij} log \frac{y_{ij}}{f_{ij}\ (\beta; x)} \tag{3}$$

Where:

- D: number of response variables (better known as components). Each response variable is a proportion, summing up to 1, hence (D-1) variables included in the model.
- n: a total of observations
- $\hat{y}_{ij}$ is the jth predicted observation for i^{th} composition.

GAMLSS model for exposure time matrices

The R package gamlss.inf [5] was used for modelling exposure time by age, which follows a mixed discrete-continuous distribution. A binary logistic model is used for the discrete part (zero or not). For the continuous part ($Y > 0$), different distributions, including Gamma, Inverse Gaussian, Inverse Gamma, Log-normal, Weibull and Pareto were tested. Below is the result of the model comparison.

Table S1: Model comparison

**Model**	**AIC**
Zero adjusted Gama model	64776.79
Zero adjusted IG model	58408.50
Zero adjusted LN model	**56596.11**
Zero adjusted WEI model	62034.31
Zero adjusted PARETO model	Not converged

Mean daily time of exposure

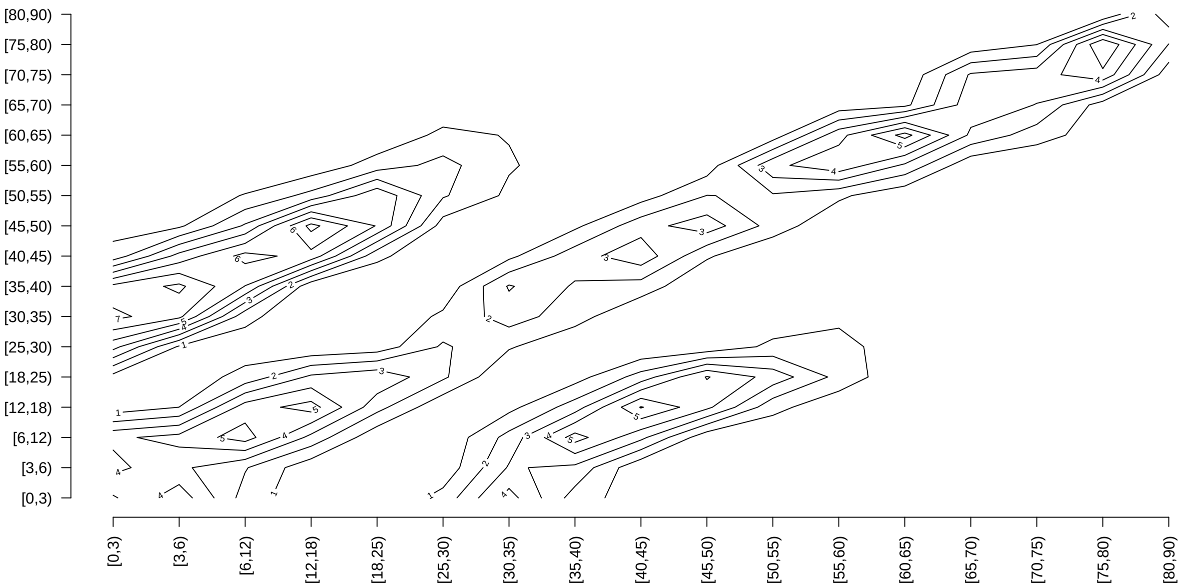

Figure S1: Mixing patterns by age based on reported presence and time spent at home

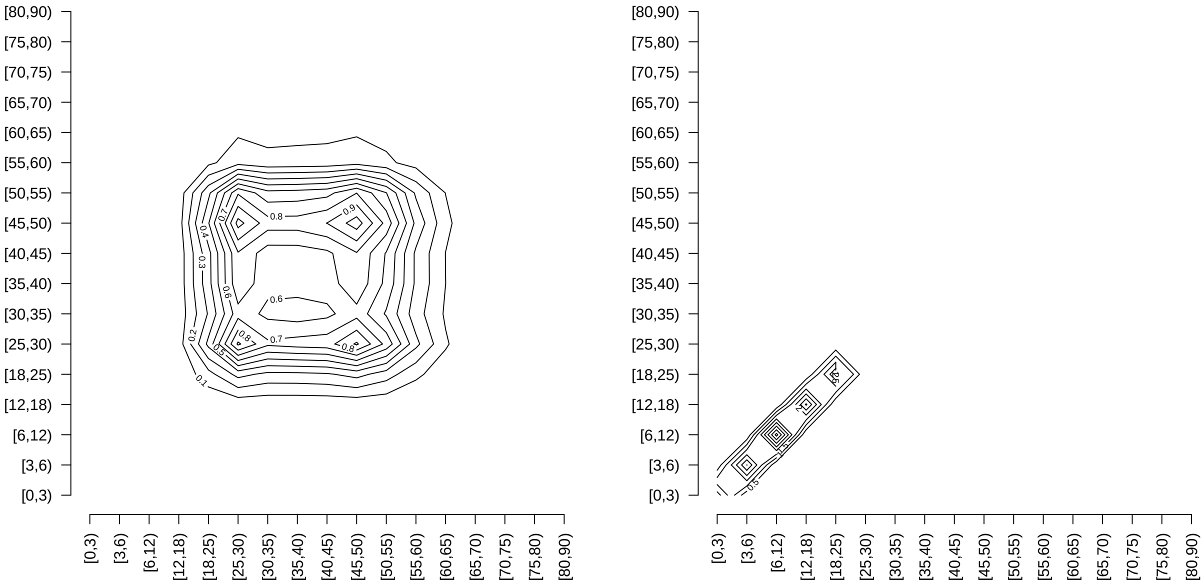

Figure S2: Mixing patterns by age based on reported presence and time spent at work (left) and school (right)

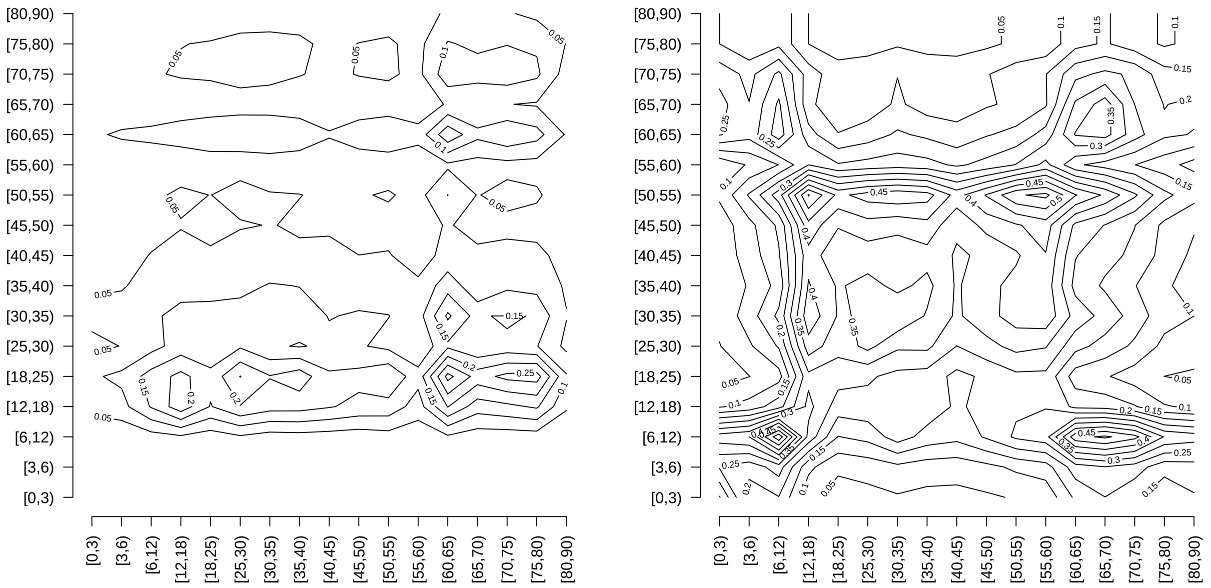

Figure S3: Mixing patterns by age based on reported presence and time spent at transport (left) and other locations (right)

Table S2: Mean daily time of exposure between people in age group i (in the rows) and people in age group j (in the columns)

	**[0;3)**	**[3;6)**	**[6;12)**	**[12;18)**	**[18;25)**	**[25;30)**	**[30;35)**	**[35;40)**	**[40;45)**	**[45;50)**	**[50;55)**	**[55;60)**	**[60;65)**	**[65;70)**	**[70;75)**	**[75;80)**	**[80;90)**
**[0;3)**	4.16	2.19	2.04	0.17	0.13	1.58	5.12	2.39	0.25	0.14	0.13	0.08	0.35	0.30	0.22	0.15	0.19
**[3;6)**	2.28	5.00	2.02	0.33	0.21	0.25	2.99	2.86	0.81	0.24	0.24	0.17	0.33	0.27	0.28	0.18	0.20
**[6;12)**	1.14	1.08	11.05	2.05	0.36	0.24	0.88	3.00	3.25	0.88	0.39	0.24	0.38	0.32	0.30	0.21	0.20
**[12;18)**	0.09	0.17	1.98	9.12	1.96	0.52	0.70	0.92	4.39	4.28	1.23	0.34	0.33	0.19	0.21	0.13	0.10
**[18;25)**	0.06	0.08	0.27	1.53	6.28	1.49	0.87	0.90	1.58	4.56	3.77	0.82	0.39	0.15	0.18	0.10	0.10
**[25;30)**	0.87	0.13	0.24	0.53	1.93	3.56	1.91	1.37	1.33	1.84	2.46	1.30	0.65	0.18	0.22	0.11	0.09
**[30;35)**	2.70	1.51	0.83	0.68	1.09	1.84	3.01	2.51	1.28	1.37	1.42	0.81	0.61	0.21	0.24	0.13	0.10
**[35;40)**	1.27	1.45	2.85	0.91	1.14	1.32	2.52	2.75	2.25	1.55	1.23	0.57	0.51	0.15	0.22	0.13	0.11
**[40;45)**	0.12	0.38	2.86	4.00	1.85	1.19	1.19	2.09	4.33	2.42	1.24	0.50	0.38	0.21	0.23	0.13	0.07
**[45;50)**	0.07	0.11	0.74	3.72	5.07	1.58	1.22	1.37	2.31	5.28	2.27	0.60	0.35	0.13	0.24	0.11	0.08
**[50;55)**	0.06	0.12	0.35	1.14	4.50	2.25	1.36	1.16	1.27	2.43	3.48	1.55	0.58	0.21	0.23	0.15	0.28
**[55;60)**	0.05	0.09	0.24	0.36	1.10	1.34	0.87	0.61	0.58	0.73	1.75	3.43	1.34	0.18	0.18	0.11	0.07
**[60;65)**	0.21	0.20	0.42	0.38	0.57	0.74	0.72	0.59	0.47	0.47	0.71	1.47	5.88	1.03	0.51	0.26	0.22
**[65;70)**	0.24	0.20	0.45	0.28	0.27	0.26	0.32	0.22	0.33	0.23	0.33	0.25	1.31	3.16	1.31	0.29	0.23
**[70;75)**	0.18	0.22	0.45	0.32	0.37	0.35	0.39	0.35	0.41	0.43	0.39	0.27	0.70	1.40	2.76	1.38	0.25
**[75;80)**	0.14	0.17	0.37	0.23	0.22	0.19	0.24	0.24	0.25	0.22	0.29	0.18	0.40	0.35	1.54	2.71	0.59
**[80;90)**	0.15	0.15	0.28	0.15	0.20	0.13	0.16	0.17	0.11	0.14	0.45	0.10	0.29	0.23	0.24	0.50	3.53

The proportion of the number of contacts and time use at different location over time settings

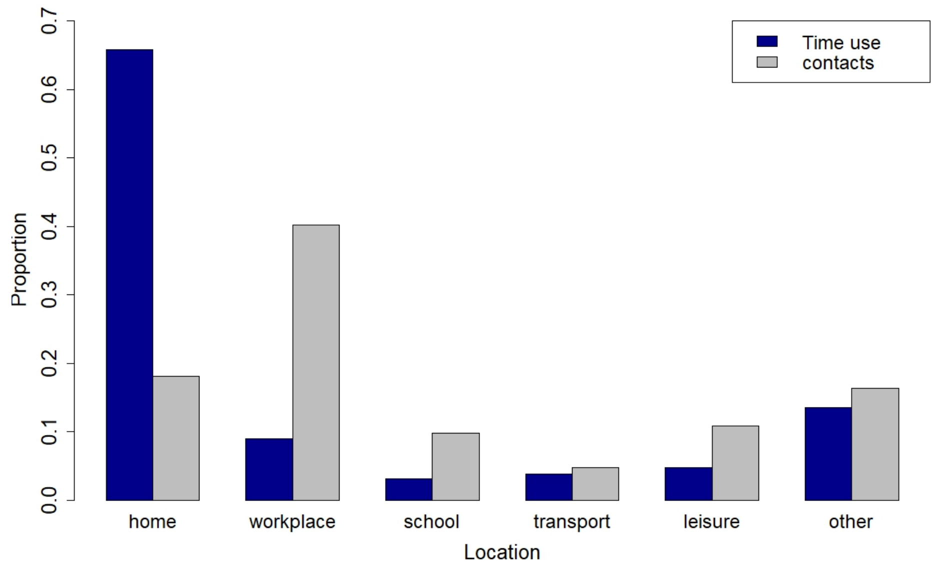

Figure S4: Proportion of number of contacts and time use

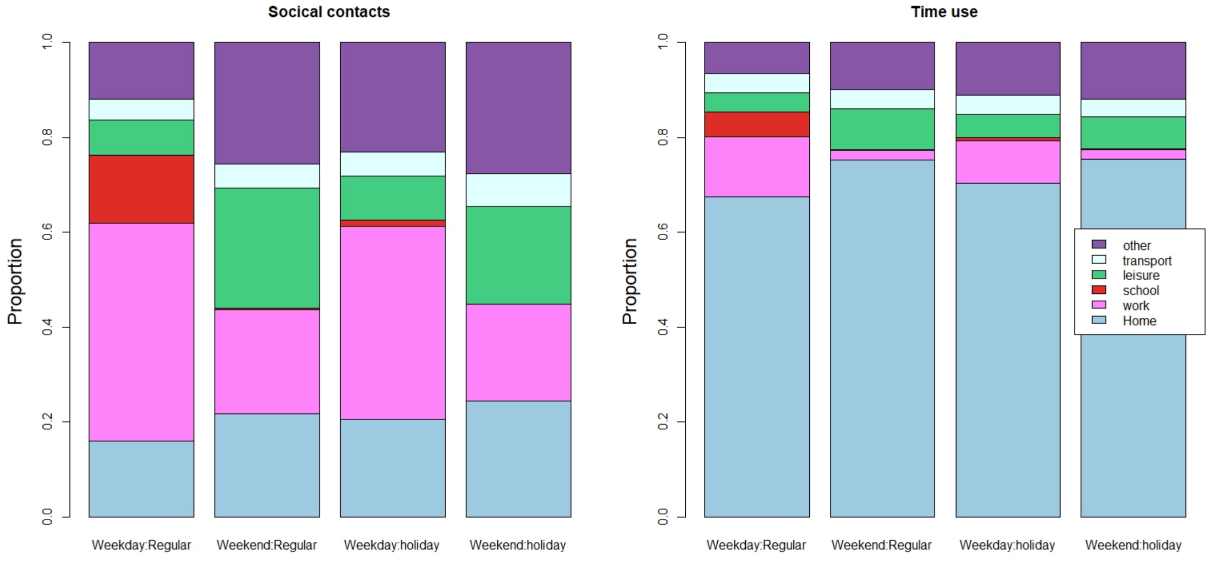

Figure S5: Proportion of number of contacts and time spent at each location in different types of day
