# Supplementary material for: Exploring human mixing patterns based on time use and social contact data and their implications for infectious disease transmission models
Source: BMC Infect Dis. 2022 Dec 19;22:954. doi: 10.1186/s12879-022-07917-y (PMC9764639; doi:10.1186/s12879-022-07917-y)
Supplement: Supplementary file 4 — Additional file 4. Number of contacts and temporal social contact matrices by distance. [file 12879_2022_7917_MOESM4_ESM.pdf]

Additional file 4: Number of contacts and temporal social contact matrices by distance

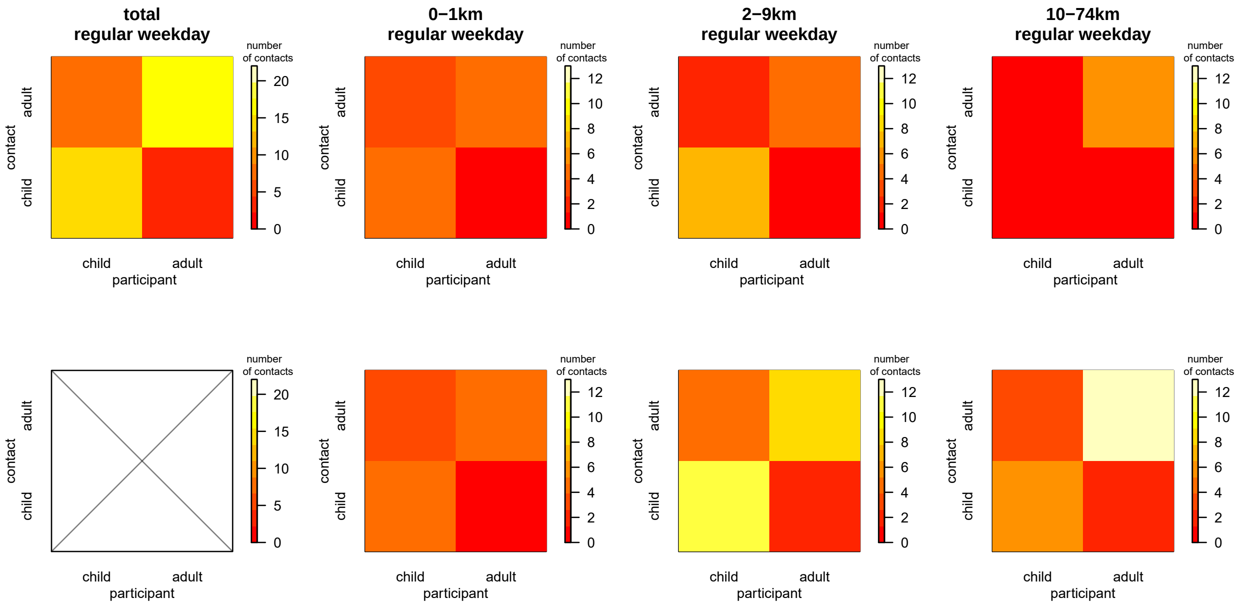

Figure S1: Social contact matrices on regular weekdays by distance, unconditional (top) and conditional (bottom) upon presence.

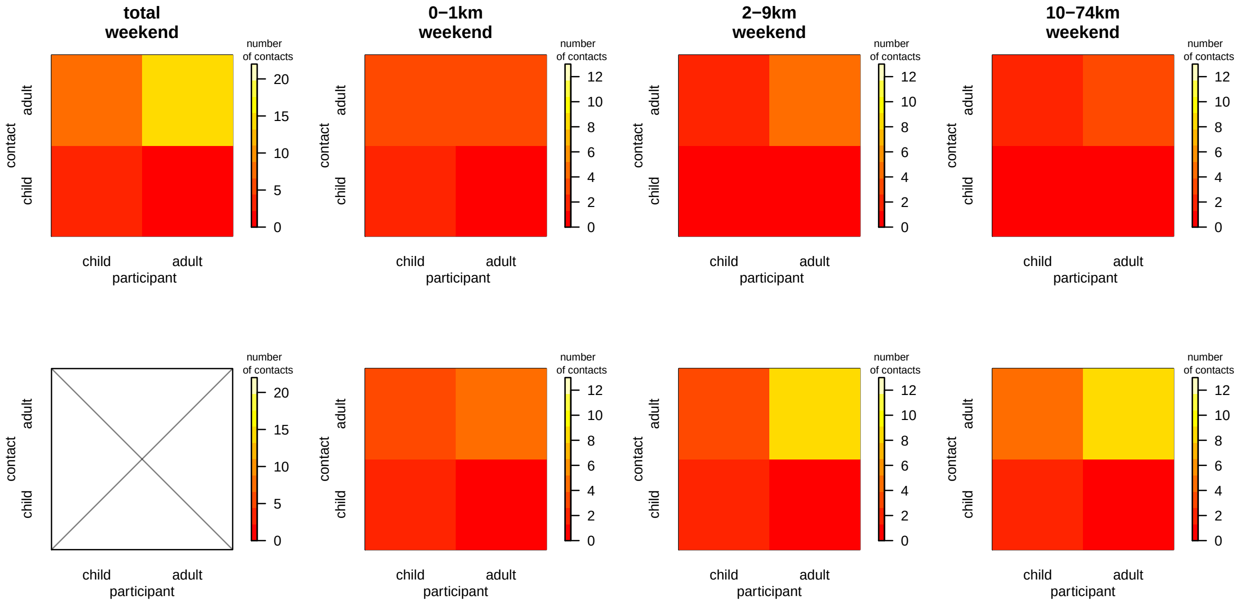

Figure S2: Social contact matrices on weekend days by distance, unconditional (top) and conditional (bottom) upon presence.
