# Supplementary material for: Exploring human mixing patterns based on time use and social contact data and their implications for infectious disease transmission models
Source: BMC Infect Dis. 2022 Dec 19;22:954. doi: 10.1186/s12879-022-07917-y (PMC9764639; doi:10.1186/s12879-022-07917-y)
Supplement: Supplementary file 5 — Additional file 5. Fitting results of social contact and exposure matrices to parvovirus-B19 and VZV sero-logical data, and ILI incidence data. [file 12879_2022_7917_MOESM5_ESM.pdf]

Additional file 5: Fitting results of social contact and exposure matrices to parvovirus-B19 and VZV sero-logical data, and ILI incidence data.

Model fit to serologial data

Table S1: MSIRWb model fits to parvovirus-B19 based on different matrices

**Contact data**	q_1	**95% CI for q_1**	ϵ	**95% CI of ϵ**	R_0	**95% CI of R_0**	**AIC**	Δ_{AIC}	**Weight**	**LER**
All contacts	0.012	[0.011;0.014]	0.006	[0.004;0.007]	2.392	[2.088;2.685]	3473.016	3.856	0.039	0.838
Close contacts	0.022	[0.019;0.024]	0.006	[0.004;0.007]	2.025	[1.827;2.207]	3470.934	1.774	0.111	0.386
Non-close contacts	0.031	[0.025;0.038]	0.006	[0.003;0.010]	3.167	[2.579;3.871]	3481.550	12.390	5.5E-04	2.691
Close < 15p	0.153	[0.096;0.232]	0.005	[0.005E-1;0.012]	2.130	[1.519;3.283]	3496.605	27.445	3.0E-07	5.960
Close > 15p	0.025	[0.022;0.029]	0.006	[0.004;0.007]	2.011	[1.835;2.197]	3469.990	0.830	0.179	0.181
Close > 1h	0.029	[0.025;0.033]	0.005	[0.004;0.006]	1.880	[1.743;2.006]	3469.976	0.816	0.180	0.178
Close > 4h	0.056	[0.0453;0.064]	0.006	[0.005;0.007]	1.895	[1.717;2.103]	**3469.158**	**0**	0.271	**0**
Linear combination of 5 locations	*	*	0.019	*	1.675	[1.552;3.401]	3476.990	7.830	0.005	1.701
**Time use data**										
Overall exposure time	0.010	[0.008;0.011]	0.007	[0.006;0.008]	2.075	[1.905;2.301]	3471.015	1.858	0.107	0.403
Linear combination of 5 locations	*	*	*	*	2.084	[1.835;2.917]	3472.352	3.194	0.055	0.694
**Combined data**										
Suitable contacts	0.097	[0.019;0.250]	0.007	[0.006;0.008]	2.152	[1.995;2.296]	3472.45	3.286	0.052	0.714
	0.129	[0.037;0.925]								

q_1 proportionality factor; [q_2] the fraction of total exposure time suitable for transmission in combined data; ϵ: waning rate
* See Appendix 6 Table 1; Δ_{AIC}: AIC difference; ER: Evidence ratio; LER: $log_{10}(ER)$.

Table S2: MSIR model fits to VZV based on different matrices

**Contact data**	q_1	**95% CI of** q_1	R_0	**95% CI of** R_0	**AIC**	Δ_{AIC}	**Weight**	**LER**
All contacts	0.045	[0.035;0.055]	7.309	[5.843;9.081]	1419.546	38.413	2.19E-09	8.341
Close contacts	0.081	[0.065;0.094]	6.389	[5.162;7.456]	1390.056	8.923	0.006	1.938
Non-close contacts	0.102	[0.081;0.188]	8.895	[7.134;16.526]	1487.455	106.322	3.93E-24	23.088
Close < 15p	0.545	[0.347;0.859]	6.510	[4.613;10.754]	1399.230	18.097	5.66E-05	3.930
Close > 15p	0.097	[0.078;0.113]	6.531	[5.290;7.692]	1388.845	7.712	0.010	1.675
Close > 1h	0.113	[0.091;0.132]	6.228	[5.114;7.392]	1385.650	4.517	0.050	0.981
Close > 4h	0.219	[0.168;0.265]	6.341	[4.907;8.003]	1386.845	5.712	0.028	1.240
Linear combination of 5 locations	*	*	7.401	[5.767;11.326]	1383.369	2.236	0.157	0.486
**Time use data**								
Overall exposure time	0.037	[0.030;0.041]	6.893	[5.717;7.778]	1384.452	3.319	0.092	0.721
Linear combination of 5 locations	*	*	19.714		1383.140	2.007	0.176	0.436
**Combined data**								
Suitable contacts	0.068	[0.061; 0.647]	**7.813**	**[6.832;8.477]**	**1381.133**	**0**	**0.481**	**0**
	0.938	[0.083; 0.952]						

q_1 proportionality factor; q_2 the fraction of total exposure time suitable for transmission in combined data;
[*] See Appendix 6 Table 1; Δ_{AIC}: AIC difference; ER: Evidence ratio; LER: $log_{10}(ER)$.

Table S3: Location-specific proportional factors in the MSIRWb and MSIR model

	**The MSIRWb model**	**The MSIR model**
**Contact data**	**fits to B19**	**fits to VZV**
Home	1.39E-11	1.10E-10
school	1.88E-02	4.07E-02
Work	2.22E-13	2.68E-10
Transport	1.78E-04	3.29E-01
General public	3.23E-13	1.66E-13
**Time use data**	**fits to B19**	**fits to VZV**
Home	1.48E-03	9.78E-13
school	1.09E-02	1.88E-01
Work	1.12E-06	6.89E-01
Transport	1.80E-05	1.19E-10
General public	1.77E-01	8.49E-02

Model fit to ILI incidence data

The model is described by the following set of differential equations:

$$\frac{dS_i}{dt} = -q_i S_i \sum_j C_{i,j} I_j$$

$$\frac{dE_i}{dt} = q_i S_i \sum_j C_{i,j} I_j - rE_i$$

$$\frac{dI_i}{dt} = rE_i - fI_i$$

$$\frac{dR_i}{dt} = fI_i$$

where:

- S_i, E_i, I_i and R_i are the number of susceptible, exposed, infected and recovered individuals respectively in age group i.
- $C_{i,j}$ is the contact rate matrices and q_i is age- dependent proportional factor; the product $q_i C_{i,j}$ translates into the average daily per capita rate at which an individual of age i makes effective contact with a person of age j.
- r is the rate at which an individual in the exposed class enters the infectious class
- f is daily rate at which infectious individuals recover and become immune.

We referred to [3, 8] and took an average latent period of 1 day (r=1) and an average infectious period of 3.8 days (f=1/3.8). We assumed that vaccination took place at the beginning of the October, with the coverage being 0.066% for 0-14 years, 5.5% for 15-19 years, 20% for 20-64 years, and 60% for 65 years and older [1]. The vaccinated people were assumed to be become immune and moved out the susceptible class immediately. The newly infected cases of the first week of the season 2010-2011, reported by GPs network [2, 7], were seeded in the model at time t=0. Given GPs network covering 1.75% of the population, we estimated infected cases from observed ILI data for the whole Belgian population. We estimated the model parameters by minimizing the sum of squared difference between the observed ILI incidence rate and the scaled model-based incidence rate, as presented in the following formula with i being age groups (0-4, 5-14, 15-19, 20-64 and $\geq$ 65) and t is the index of week from 1 to 52 in the season 2010-2011. α is a scaling factor to account for several issues, e.g. consultation rate at GPs, mis-specification of model parameters...

$$\sum_i \sum_t \left[\frac{I_{i,t}}{N_i} - \alpha \frac{\hat{I_{i,t}}}{N_i} \right]^2$$

The accuracy of the model is measured by the mean absolute error (MAE), where I_t and $\hat{I}_t$ are observed number of cases and estimated number of cases at time t, respectively.

$$MAE = mean(\left| I_t - \hat{I}_t \right|)$$

Table S4: Model fits to ILI incidence data based on different matrices

**Contact data**	$q_{1.1}$**(95% CI)**	$q_{1.2}$**(95% CI)**	$q_{1.3}$**(95% CI)**	$q_{1.4}$**(95% CI)**	$q_{1.5}$**(95% CI)**	q_2**(95% CI)**	**alpha(95%)**	**R0(95%)**	**LS value**
All contacts	0.137(0.124-0.158)	0.09 (0.079-0.098)	0.131(0.114-0.148)	0.090(0.084-0.096)	0.120(0.086-0.122)		0.265(0.265-0.268)	2.077(1.881-2.470)	8.66e-5
Non-close contacts	0.420(0.326-0.546)	0.207(0.198-0.394)	0.257(0.149-0.451)	0.165(0.0910-.329)	0.005(0.003-0.009)		0.269(0.261-0.270)	3.471(2.130-3.674)	9.32e-5
Close contacts	0.212(0.162-0.325)	0.167(0.104-0.213)	0.274(0.210-0.421)	0.204(0.125-0.381)	0.242(0.210-0.391)		0.265(0.201-0.269)	1.510(1.458-1.637)	8.47e-5
Close > 15p	0.254(0.135-0.342)	0.192(0.124-0.236)	0.327(0.213-0.548)	0.250(0.215-0j.451)	0.301(0.281-0.428)		0.265(0.260-0.268)	1.512(1.457-1.742)	8.38e-5
Close > 1h	0.303(0.254-0.465)	0.230(0.201-0.342)	0.422(0.327-0.479)	0.332(0.312-0.541)	0.293(0.213-0.415)		0.266(0.262-0.269)	1.429(1.324-1.543)	8.28e-5
Close > 4h	0.544(0.326-0.642)	0.438(0.218-0.526)	0.807(0.231-0.902)	0.673(0.429-0.791)	0.514(0.491-0.615)		0.266(0.260-0.291)	1.318(1.131-1.635)	8.34e-5
**Time use data**									
Overall exposure time	0.097(0.081-0.102)	0.076(0.069-0.812)	0.119(0.107-0.129)	0.08(0.077-0.082)	0.149(0.94-0.198)		0.264(0.261-0.2661)	1.630(1.401-1.722)	8.37e-5
**Combined data**									
Suitable contacts	0.284(0.258-0.497)	0.21(0.199-0.397)	0.322(0.303-0.619)	0.218(0.212-0.418)	0.336(0.250-0.638)	0.478(0.216-0.502)	0.264(0.262-0.266)	1.441(1.430-1.446)	8.43e-5

$q_{1.1}$-$q_{1.5}$: The age-dependent proportionality factors;
q_2: The fraction of total exposure time suitable for transmission in combined data;
α: the scaling factor

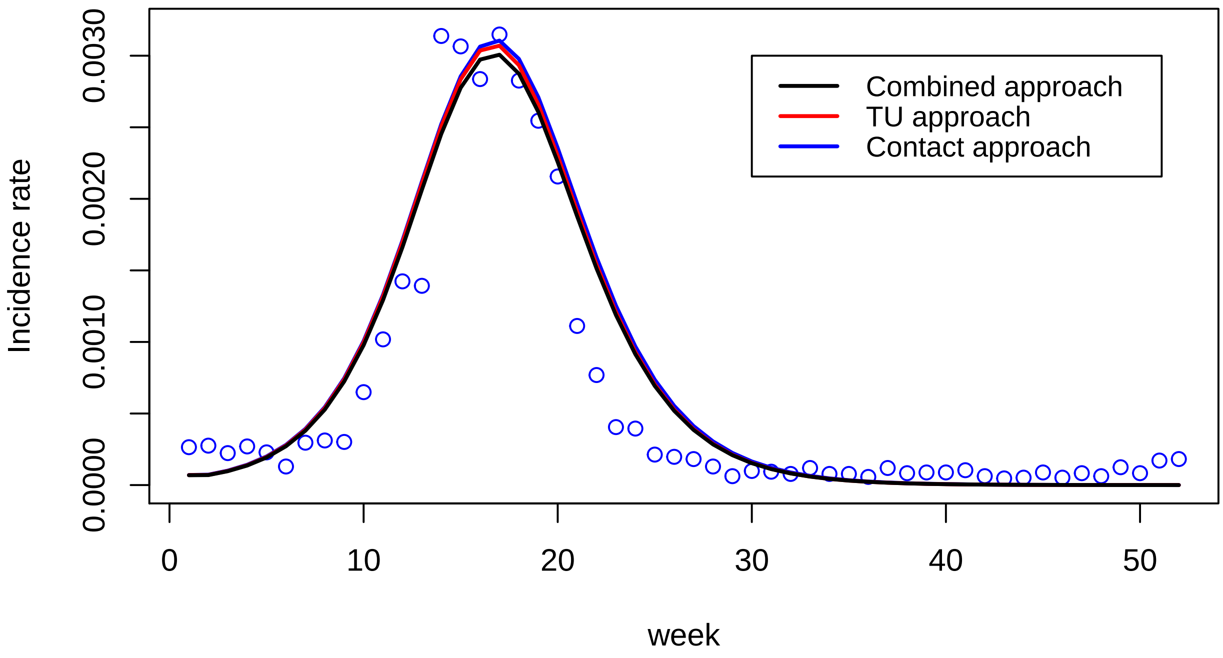

Figure S1: The fit to ILI incidence data.
