# Supplementary material for: Exploring human mixing patterns based on time use and social contact data and their implications for infectious disease transmission models
Source: BMC Infect Dis. 2022 Dec 19;22:954. doi: 10.1186/s12879-022-07917-y (PMC9764639; doi:10.1186/s12879-022-07917-y)
Supplement: Supplementary file 6 — Additional file 6. Reference list for additional files. [file 12879_2022_7917_MOESM6_ESM.pdf]

References

[1] P. Beutels, Y. Vandendijck, L. Willem, N. Goeyvaerts, A. Blommaert, K. Van Kerckhove, J. Bilcke, G. Hanquet, P. Neels, N. Thiry, et al. Seasonal influenza vaccination: prioritizing children or other target groups? part ii: cost-effectiveness analysis. *Health Technology Assessment (HTA) Brussels: Belgian Health Care Knowledge Centre (KCE)*, 2013.

[2] K. Bollaerts, J. Antoine, V. Van Casteren, G. Ducoffre, N. Hens, and S. Quoilin. Contribution of respiratory pathogens to influenza-like illness consultations. *Epidemiology & Infection*, 141(10):2196–2204, 2013.

[3] N. Goeyvaerts, L. Willem, K. Van Kerckhove, Y. Vandendijck, G. Hanquet, P. Beutels, and N. Hens. Estimating dynamic transmission model parameters for seasonal influenza by fitting to age and season-specific influenza-like illness incidence. *Epidemics*, 13:1–9, 2015.

[4] M. Schomaker and C. Heumann. Bootstrap inference when using multiple imputation. *Statistics in medicine*, 37(14):2252–2266, 2018.

[5] M. Stasinopoulos, M. Enea, and R. A. Rigby. Zero adjusted distributions on the positive real line. *http://www.gamlss.com*, 2017.

[6] M. Tsagris and C. Stewart. A dirichlet regression model for compositional data with zeros. *Lobachevskii Journal of Mathematics*, 39(3):398–412, 2018.

[7] V. Van Casteren, K. Mertens, J. Antoine, S. Wanyama, I. Thomas, and N. Bossuyt. Clinical surveillance of the influenza a (h1n1) 2009 pandemic through the network of sentinel general practitioners. *Archives of Public Health*, 68(2):1–6, 2010.

[8] E. Vynnycky, R. Pitman, R. Siddiqui, N. Gay, and W. J. Edmunds. Estimating the impact of childhood influenza vaccination programmes in england and wales. *Vaccine*, 26(41):5321–5330, 2008.
